# Assessing the potential for competition between Pacific Halibut (*Hippoglossus stenolepis*) and Arrowtooth Flounder (*Atheresthes stomias*) in the Gulf of Alaska

**DOI:** 10.1371/journal.pone.0209402

**Published:** 2018-12-18

**Authors:** Cheryl L. Barnes, Anne H. Beaudreau, Mary E. Hunsicker, Lorenzo Ciannelli

**Affiliations:** 1 College of Fisheries and Ocean Sciences, University of Alaska Fairbanks, Juneau, Alaska, United States of America; 2 Fish Ecology Division, Northwest Fisheries Science Center, National Marine Fisheries Service, National Oceanic and Atmospheric Administration, Newport, Oregon, United States of America; 3 College of Earth, Ocean, and Atmospheric Sciences, Oregon State University, Corvallis, Oregon, United States of America; Texas A&M University, UNITED STATES

## Abstract

Pacific Halibut (*Hippoglossus stenolepis*) support culturally and economically important fisheries in the Gulf of Alaska, though recent decreases in mean size-at-age have substantially reduced fishery yields, generating concerns among stakeholders and resource managers. Among the prevailing hypotheses for reduced size-at-age is intensified competition with Arrowtooth Flounder (*Atheresthes stomias*), a groundfish predator that exhibited nearly five-fold increases in biomass between the 1960s and mid-2010s. To assess the potential for competition between Pacific Halibut and Arrowtooth Flounder, we evaluated their degree of spatiotemporal and dietary overlap in the Gulf of Alaska using bottom trawl survey and food habits data provided by the Alaska Fisheries Science Center (NOAA; 1990 to 2017). We restricted analyses to fish measuring 30 to 69 cm fork length and used a delta modeling approach to quantify species-specific presence-absence and catch-per-unit-effort as a function of survey year, tow location, depth, and bottom temperature. We then calculated an index of spatial overlap across a uniform grid by multiplying standardized predictions of species’ abundance. Dietary overlap was calculated across the same uniform grid using Schoener’s similarity index. Finally, we assessed the relationship between spatial and dietary overlap as a measure of resource partitioning. We found increases in spatial overlap, moving from east to west in the Gulf of Alaska (eastern: 0.13 ± 0.20; central: 0.21 ± 0.11; western: 0.31 ± 0.13 SD). Dietary overlap was low throughout the study area (0.13 ± 0.20 SD). There was no correlation between spatial and dietary overlap, suggesting an absence of resource partitioning along the niche dimensions examined. This finding provides little indication that competition with Arrowtooth Flounder was responsible for changes in Pacific Halibut alHHsize-at-age in the Gulf of Alaska; however, it does not rule out competitive interactions that may have affected resource use prior to standardized data collection or at different spatiotemporal scales.

## Introduction

Pacific Halibut (*Hippoglossus stenolepis*) is a large-bodied flatfish that is ecologically important as an apex predator in the Gulf of Alaska [1] and has supported commercial, recreational, and subsistence fisheries for well over a century [2–3]. However, decreases in spawning stock biomass and mean size-at-age between the 1970s and mid-2000s [3–5] have raised concerns among stakeholders and resource managers regarding the long-term productivity of the stock. In fact, declines in size-at-age have been identified as the most important driver of recent trends in stock dynamics for Pacific Halibut, especially in the Gulf of Alaska [6]. Loher [7] described a suite of potentially interacting mechanisms that could be responsible for reduced size-at-age of Pacific Halibut. These included shifts in metabolic demands or efficiencies due to environmental variation, decreases in prey quality or availability, cumulative effects of size-selective fishing, a release of predation pressure on smaller size classes, density-dependent effects due to intraspecific competition, and intensified interspecific competition with Arrowtooth Flounder (*Atheresthes stomias*). Arrowtooth Flounder is a flatfish predator with similar niche requirements that has displayed nearly five-fold increases in biomass over the same time period of observed decreases in halibut size-at-age [8].

A number of studies have been carried out to test the potential mechanisms for decreased Pacific Halibut size-at-age described by Loher [7]. Clark *et al*. [9] found that recent decreases in halibut growth followed a shift in the Pacific Decadal Oscillation between 1976 and 1977, suggesting negative effects of warming temperatures. A subsequent study by Clark and Hare [4] assessed changes in Pacific Halibut size-at-age over a longer timeframe and found relatively small size-at-age in the early 1920s, subsequent increases to a peak around 1970, and decreases to historical size-at-age by the mid to late 1990s. These authors attributed decreases in growth to density dependent effects associated with elevated stock sizes. Recent experiments conducted by Planas [10] have demonstrated positive effects of temperature on somatic growth for captive juveniles. Holsman *et al*. [11] found a similar relationship in the Gulf of Alaska, attributing higher potential growth in juvenile halibut to increased metabolic demands and foraging rates in warmer waters. Prey quality has also been suggested as affecting halibut growth and subsequent size-at-age. For example, Webster [12] found that ‘fast’ growing halibut (*i*.*e*., younger fish from a specific size class) exhibited more benthic-associated diets with prey from higher trophic levels, whereas ‘slow’ growing halibut (*i*.*e*., older fish from the same size class) consumed more, lower trophic-level fishes. Another study by Sullivan [13] used population modeling techniques to test the cumulative effects of size-selective fishing on halibut size-at-age. She found that harvest-based removals explained 30 to 65% of within-regional variation in size-at-age throughout the Gulf of Alaska.

Despite these efforts, our understanding about drivers of change in Pacific Halibut size-at-age is incomplete [3], as many of the alternative hypotheses posed by Loher [7] have not yet been fully explored. This includes the hypothesis that competitive interactions between Pacific Halibut and Arrowtooth Flounder have intensified in the Gulf of Alaska, resulting in decreased growth rates and subsequent declines in halibut size-at-age. At present, our understanding about this particular mechanism is based on a negative correlation between Pacific Halibut growth and Arrowtooth Flounder biomass [13]. Yet inferring the potential for competition among wild fish populations requires three conditions apart from opposite population trajectories: high spatiotemporal overlap, high dietary overlap, and evidence of resource limitation [14]. These criteria, if met, would suggest that competition is ongoing or is likely to take place in the future. To infer past competition between large-bodied, highly mobile marine species, we employ the theory of resource partitioning, which states that competing species must differentiate their resource use along one or more niche dimensions in order to coexist (*e*.*g*., [15–18]).

There are three niche dimensions over which species commonly partition resources to alleviate competitive pressures: space, time, and food [18]. In terms of space, individuals may occupy different microhabitats or utilize different depth ranges while foraging on similar prey within the same environment (*e*.*g*., [19]). Temporal segregation may take place in the form of occupying the same location at different points in the season or at different times of day [17]. If found in the same place at the same time, competing species must differentiate the types or sizes of prey consumed, a tactic common in marine systems [16–18]. Each of these scenarios reflects an actual niche that is smaller than the virtual (*i*.*e*., ‘pre-competitive’) niche of one or both species [20]. This concept of resource partitioning would be illustrated by a negative relationship between spatiotemporal overlap and dietary overlap at scales relevant to the movements and foraging activities of both potential competitors. In other words, we would expect dietary overlap to decrease with increasing spatiotemporal overlap and vice versa. If a positive relationship between spatiotemporal overlap and dietary overlap were observed instead, we might infer that competition is in its early stages, is ongoing, or may take place in the future as resources become limiting [14].

We quantified the relationship between spatiotemporal and dietary overlap for Pacific Halibut and Arrowtooth Flounder to assess their degree of resource partitioning along multiple niche dimensions in the Gulf of Alaska using long-term, broad-scale catch and diet data collected by the Alaska Fisheries Science Center (AFSC). Based upon species-specific physiological constraints, we hypothesized that spatial overlap would be greatest at depth and thermal ranges shared by the two species (e.g., 150 to 200 m and 3 to 9°C) [21–23]. We also expected spatial overlap to be greatest during earlier survey years (*e*.*g*., 1996 to 2001), when estimates of Pacific Halibut spawning stock biomass were at their highest [3]. We hypothesized that dietary compositions would be most similar for relatively large (*i*.*e*., 60 to 69 cm) size classes of Pacific Halibut and Arrowtooth Flounder, whose diets consist of greater proportions of fish prey [1,24–26]. We also expected dietary overlap to be greatest in the western Gulf of Alaska, where biodiversity is relatively low [21], and vary by year as preferred prey populations fluctuated with changing environmental conditions. Finally, we postulate a negative relationship between spatial overlap and dietary overlap (*i*.*e*., evidence of resource partitioning) if competition with Arrowtooth Flounder served as a mechanism for decreased growth and, therefore, size-at-age of Pacific Halibut in the Gulf of Alaska.

## Methods

### Overview

We used fishery-independent bottom trawl survey and food habits data collected by the Alaska Fisheries Science Center (AFSC, National Oceanic and Atmospheric Administration; see [27] for methods) to assess the relationship between spatiotemporal overlap (referred to simply as spatial overlap forward going) and dietary overlap for Pacific Halibut and Arrowtooth Flounder in the Gulf of Alaska. Species-specific distributions and abundances were first modeled as a function of spatiotemporal and environmental covariates. Standardized abundances for each species were then multiplied to derive an index of spatial overlap across a uniform grid system. Dietary overlap was calculated across the same gridded system using an index of similarity that incorporated proportions of prey by weight data. We tested the correlation between spatial and dietary overlap as a measure of resource partitioning. All data analyses were conducted using the statistical programming environment R [28]. Applicable code can be found at: https://github.com/cheryl-barnes/ResourcePartitioning.git.

### Data description

**Fig 1:**
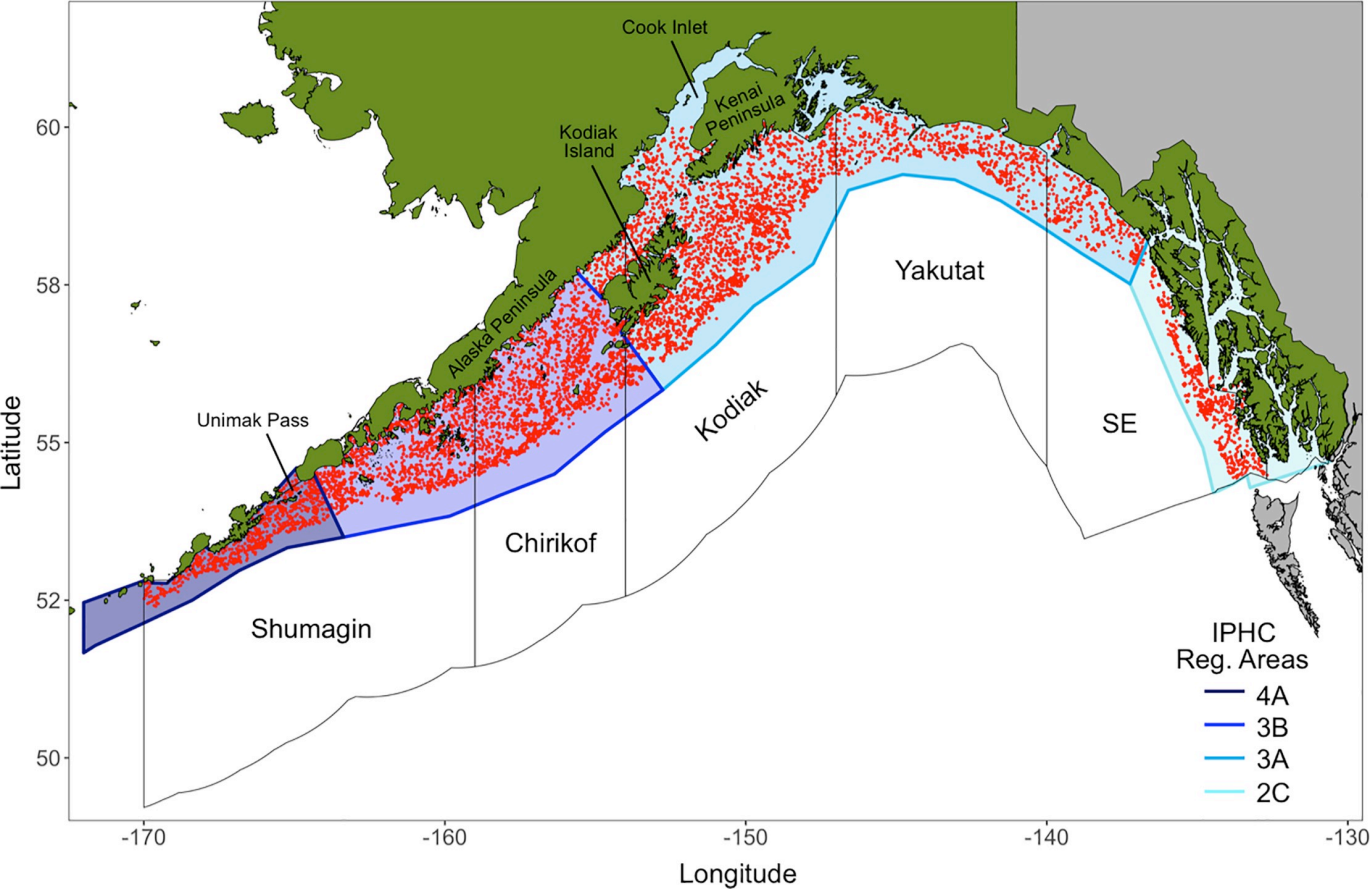
Map of bottom trawl survey area (Alaska Fisheries Science Center, NOAA; 1990 to 2017). Red dots indicate individual tow locations throughout the Gulf of Alaska. Unfilled polygons outlined in black denote Shumagin, Chirikof, Kodiak, Yakutat, and Southeastern (SE) statistical areas defined by International North Pacific Fisheries Commission (INPFC). Blue-shaded polygons illustrate International Pacific Halibut Commission (IPHC) regulatory areas 4A, 3B, 3A, and 2C.

Bottom trawl surveys were carried out by the AFSC’s Resource Assessment and Conservation Engineering (RACE) Division using a stratified random sampling design that spanned the International North Pacific Fisheries Commission (INPFC) statistical areas in the Gulf of Alaska (*i*.*e*., Shumagin, Chirikof, Kodiak, Yakutat, and Southeastern) [29]. These statistical areas generally correspond to International Pacific Halibut Commission (IPHC) regulatory areas 4A, 3B, 3A, and 2C [30] (Fig 1). Surveys were completed triennially from 1990 to 1999 and biennially from 2001 to 2017. However, the Yakutat and Southeastern INPFC areas (IPHC area 2C and the eastern half of 3A) were not surveyed in 2001. Surveys were systematically conducted from west to east, confounding time and space. The Shumagin INPFC area (IPHC area 4A) was typically sampled in mid May and the Southeastern INPFC area (IPHC area 2C) was typically sampled in mid to late July. Individual tows were approximately 15 minutes in duration at a continuous vessel speed of 5.6 m per sec [29]. Bottom trawl survey data are publicly available online at https://www.afsc.noaa.gov/RACE/groundfish/survey_data/data.htm.

All fishes were identified to species and enumerated for calculations of catch-per-unit-effort (CPUE; number of fish per hectare) [29]. Capture date, location (latitude and longitude), depth (m), and bottom temperature (°C) were recorded whenever possible. Fork length measurements (cm) were also recorded for up to 200 randomly selected fish per species per haul. Up to five fish lacking any signs of net feeding (i.e., consuming prey items while inside the trawl net) or regurgitation were sampled for diets from each haul and size category: < 31 cm, 31 to 50 cm, 51 to 70 cm, and > 70 cm [30]. Signs of net feeding and regurgitation used to discard samples were the presence of prey in the mouth or gills or a flaccid stomach observed upon dissection. Fish exhibiting signs of regurgitation were discarded and replaced with fish that had non-empty stomachs [31]. Stomach fullness was approximated (1: empty; 2: traces of prey; 3: < 25% full; 4: 25 to 49% full; 5: 50 to 74% full; 6: 75 to 100% full; 7: distended) and prey from non-empty stomachs were identified to the lowest possible taxonomic group, weighed (0.001 g), and measured wherever possible. Food habits data were provided by the AFSC’s Resource Ecology and Ecosystem Modeling (REEM) Program for survey years between 1990 and 2013, though fishes caught in the Yakutat INPFC area (eastern half of IPHC area 3A) were not subsampled in 1996, 1999, or 2001 and those caught in the Southeastern INPFC area (IPHC area 2C) were not subsampled prior to 2003. The diet data used in this study are publicly available at https://access.afsc.noaa.gov/REEM/WebDietData/DietDataIntro.php.

### Spatial distributions and spatial overlap

**Fig 2:**
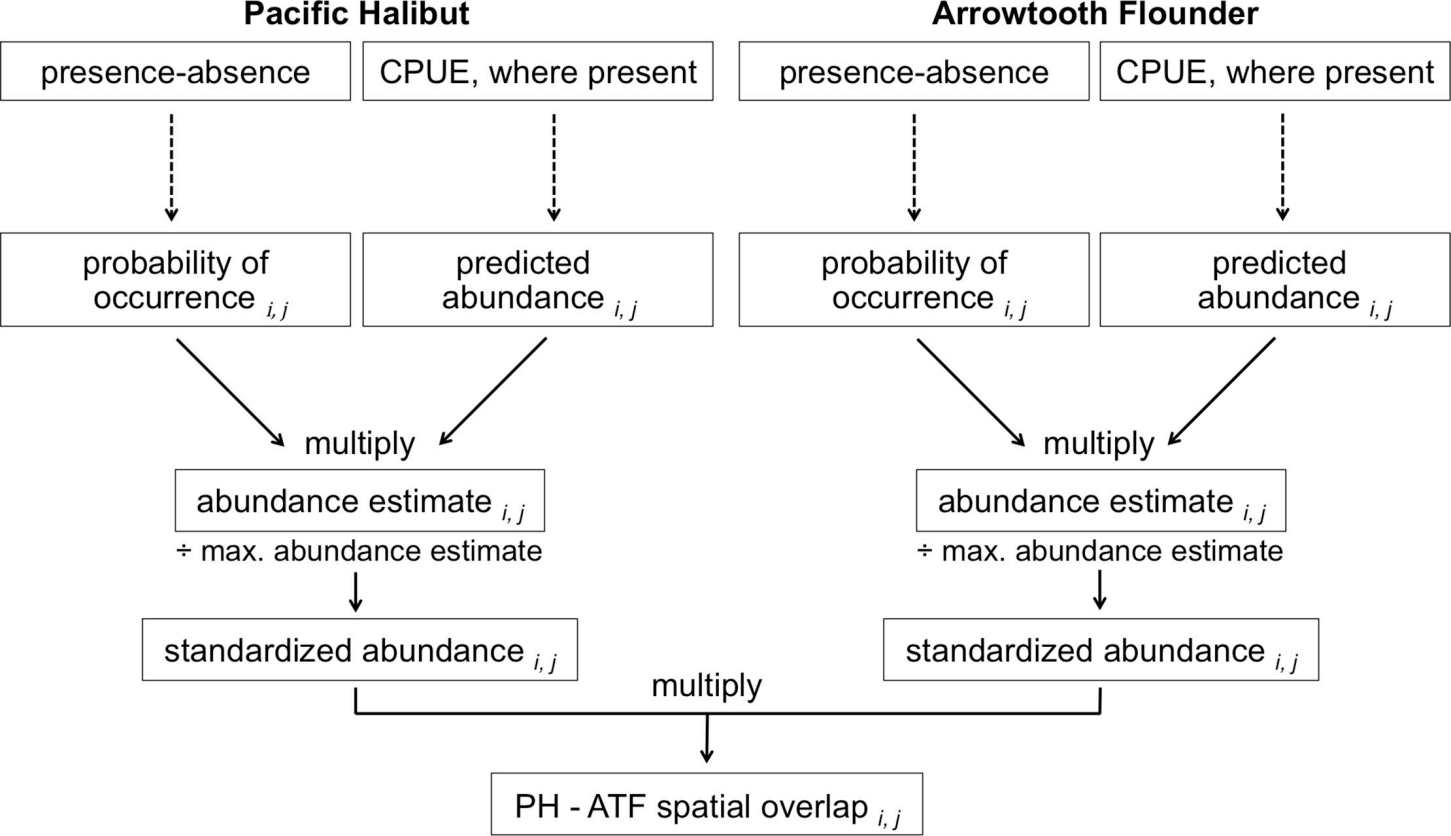
Analytical framework used to quantify spatial overlap between Pacific Halibut and Arrowtooth Flounder. First, bottom trawl survey data from the Gulf of Alaska and generalized additive models were used to separately quantify presence-absence and catch-per-unit-effort (CPUE; number per ha) as a function of survey year, tow location (latitude, longitude), depth, and bottom temperature. Model results were used to estimate the probability of occurrence and predicted abundance for Pacific Halibut or Arrowtooth Flounder in each combination of survey year *i* and uniform grid cell *j*. These predictions were multiplied to estimate abundance, which was then standardized by dividing each survey year-grid cell value by that species’ maximum across all years. Finally, standardized abundances for Pacific Halibut and Arrowtooth Flounder were multiplied to approximate spatial overlap in each survey year and grid cell. Analytical methods were modified from those described by Hunsicker *et al*. [32] and Shelton *et al*. [33].

We used a multi-stage modeling approach, modified from Hunsicker *et al*. [32] and Shelton *et al*. [33], to quantify spatial overlap between Pacific Halibut and Arrowtooth Flounder in the Gulf of Alaska (Fig 2). The smallest fish sampled were predominately Arrowtooth Flounder and the largest individuals were exclusively Pacific Halibut. Because size is another dimension over which resource partitioning can take place [17], we accounted for size by restricting analyses to fish measuring between 30 and 69 cm fork length. Based on available age-length relationships, this restricted size range corresponds to Pacific Halibut ≤ 7 yr [13] and Arrowtooth Flounder ≥ 3 yr [34]. These size restrictions equated to 67.6% of Pacific Halibut and 75.7% of Arrowtooth Flounder subsampled for measurements.

To account for over-dispersion resulting from a zero-inflated data set, we used a delta (*i*.*e*., hurdle) model consisting of two parts [35–37]. First, we used generalized additive models (GAMs) with a logit link function to model the binary response of presence (1) or absence (0) as a function of survey year, tow location (*i*.*e*., latitude and longitude), depth (m), and bottom temperature (°C) (‘mgcv’ package in R) [38]. The full model formulation for the probability that species *s* was present in haul *h* (assuming a binomial distribution with an expected value of *μ*) was:

$$log(\mu_{h,s})=y_{i}+f_{1}(\emptyset_{h},\lambda_{h})+f_{2}(z_{h})+f_{3}(T_{h})$$

$$E(p_{h,s})=\mu_{h,s},\;p_{h,s}∼B(1,\mu_{h,s}),\;var(p_{h,s})∼\mu_{h,s}(1-\mu_{h,s})$$

*f* indicates bivariate (1: longitude ∅, latitude *λ*) or univariate (2: depth *z* and 3: bottom temperature *T*) smoothing functions and *y* represents survey year *i*. Next, we used GAMs with a Gaussian distribution and identity link to model log-transformed CPUE (number of fish per hectare) data, where either Pacific Halibut or Arrowtooth Flounder were present in a haul (‘mgcv’ package in R) [38]. Log-transformations are commonly used with CPUE data to reduce skewness resulting from a small number of stations with unusually large catch rates [39]. Because fork lengths were not recorded for all fishes caught, we adjusted haul-specific CPUE estimates by multiplying the proportion of individuals measuring between 30 and 69 cm in subsamples by total CPUE for each haul. Just as with presence-absence, CPUE was modeled as a function of survey year, tow location, depth, and bottom temperature. The full model formulation was: *x*_*h*,*s*_ = *y*_*i*_+*f*_1_(∅_*h*_,*λ*_*h*_)+*f*_2_(*z*_*h*_)+*f*_3_(*T*_*h*_)+*ε*_*h*,*s*_, where x denotes the natural log of CPUE for species *s* in haul *h*.

Separately modeling presence-absence and CPUE for hauls with positive catches *e*.*g*., [40–42] allows for unique responses of species distribution and abundances to model covariates. Though generalized linear mixed models (GLMMs) are more commonly used with the delta modeling approach, we elected for the greater flexibility of GAMs given that species-habitat associations are likely nonlinear [43]. We did not include a spatial autocorrelation term because residuals were not correlated at the scale of our predictions (*i*.*e*., 100 km). Though depth and bottom temperature were correlated (r_8634_ = - 0.41, t_8634_ = - 42.08, p < 0.001), we were specifically interested in the individual effects of each of these covariates on probability of occurrence and CPUE of Pacific Halibut and Arrowtooth Flounder in the Gulf of Alaska.

To ensure that all GAMs were based on the same suite of data, we excluded tows with missing depths or bottom temperatures. Survey year was treated as a fixed factor and the amount of smoothing for nonparametric terms was determined within each model using generalized cross-validation (GCV) [44]. Smoothing functions for bottom temperature were limited to four knots to avoid over-fitting. However, we did not constrain the degree of smoothing for depth or the bivariate location term (longitude, latitude), enabling detection of patterns in space use that may vary at higher orders. Once full models were constructed, we used the dredge function from the ‘MuMIn’ package in R [45] to generate a comprehensive suite of alternative models for each combination of species and response variable (presence-absence and CPUE). We then selected best-fit models using Akaike Information Criterion (AIC), which balances model fit and model complexity [46]. Partial effects of each model covariate were interpreted to help distinguish between environmental drivers of spatial distributions and potential influences of competition.

To calculate spatial overlap from model results, we had to first estimate the probability of occurrence and predicted abundance of Pacific Halibut and Arrowtooth Flounder across a uniform grid system spanning the spatial extent of the bottom trawl survey. This uniform grid allowed for predictions at a finite number of locations (*i*.*e*., latitude and longitude coordinates pertaining to individual grid cell centers), established standardized units of area for grouping diet data, and ensured that estimates of spatial and dietary overlap were directly comparable to one another in time and space–a necessary component for assessing the degree of resource partitioning between two potential competitors. We constructed the grid using a Universal Transverse Mercator (UTM) coordinate system before projecting to decimal degrees (‘PBSmapping’ [47], ‘rgdal’ [48], ‘rgeos’ [49], and ‘sp’ [50] packages in R). Mean depths and mean bottom temperatures for unique combinations of survey year and grid cell were used as input data for estimating probabilities of occurrence and predicted abundances from best-fit GAMs. We then multiplied the probability of occurrence (${PO}_{s_{i,j}}$) and predicted abundance (${PA}_{s_{i,j}}$) in each survey year *i* and grid cell *j* to estimate overall abundance ($A_{s_{i,j}}$) for each species *s* ($A_{s_{i,j}}={PO}_{s_{i,j}}*{PA}_{s_{i,j}}$). Abundance estimates were standardized by dividing each survey year-grid cell value by the maximum predicted abundance estimate for a given species, across all survey years and grid cells ($std\;A_{s_{i,j}}=A_{s_{i,j}}/{maxA}_{s}$). We elected to use the species-specific maximum predicted abundance because it produced the desired range of values (*i*.*e*., 0 to 1) for use in calculating spatial overlap. Additionally, standardizing by the species-specific mean or median resulted in nearly identical patterns (though on different scales), demonstrating the robustness of this approach (S1 Fig). Grid cells resulting in standardized abundances less than 0.25 for both Pacific Halibut and Arrowtooth Flounder suggested poor habitat suitability and were excluded from further analyses. The three grid cells eliminated were among the deepest sampled. Finally, we multiplied standardized abundance estimates to approximate spatial overlap (*S*_*i*,*j*_) between Pacific Halibut (*PH*) and Arrowtooth Flounder (*ATF*) throughout the Gulf of Alaska ($S_{i,j}=std\;A_{{PH}_{i,j}}*std\;A_{{ATF}_{i,j}}$). Spatial overlap was estimated for 681 unique combinations of survey year and grid cell, with a possible range of values from 0 (no overlap) to 1 (complete overlap).

Year-specific estimates of spatial overlap were averaged within each grid cell to illustrate overall approximations of spatial overlap at each location. We assessed regional and temporal changes in spatial overlap using an analysis of covariance (ANCOVA), treating INPFC statistical area or IPHC regulatory area as the fixed effect and year as the model covariate. Significance was determined using an α set to 0.1. Tukey Honest Significant Differences (Tukey HSD) tests (‘stats’ package in R [28]) were used to make post hoc comparisons when significant effects of area were identified.

### Diet compositions and dietary overlap

As with our spatial modeling, we limited diet analyses to fish measuring 30 to 69 cm fork length. This reduced ontogenetic variation in diet compositions and increased comparability between the two species. These size restrictions equated to 60.2% and 72.2% of the non-empty stomachs sampled for Pacific Halibut and Arrowtooth Flounder, respectively. We calculated proportions of prey by weight (*W*) for each prey taxon *t* found in the stomach of predator species *s* in survey year *i* and grid cell *j*, given the following equation (modified from Chipps and Garvey [51]):

$$W_{t,s,i,j}=\frac{W_{t,s,i,j}}{\sum_{t=1}^{Q}W_{t,s,i,j}},\;\mathrm{where}$$

*Q* is the total number of prey taxa observed. Proportions were also calculated for distinct size classes (*i*.*e*., 30 to 39 cm, 40 to 49 cm, 50 to 59 cm, 60 to 69 cm) to qualitatively assess ontogenetic variation in diets. We elected to calculate proportions of prey by weight instead of some other dietary index (*e*.*g*., proportion of prey by number, frequency of occurrence) because we were interested in comparing the relative contributions of various prey taxa to the diets of Pacific Halibut and Arrowtooth Flounder [52]. To provide additional comparisons of dietary niche breadth, we constructed species-specific rarefaction curves (S2 Fig), computed the Shannon-Weaver index of diversity (H’), and calculated Pielou’s index for evenness (J’) using the ‘vegan’ package in R [53].

Schoener’s index [54–55] of dietary overlap provides simple and robust calculations that are free from assumptions about the nature of competition [56–57], thus we quantified dietary overlap as follows:

$$D_{i,j}=1-\frac{1}{2}\sum_{t}^{Q}\left|W_{{PH}_{t,i,j}}-W_{{ATF}_{t,i,j}}\right|,\;\mathrm{where}$$

$W_{{PH}_{t}}$ and $W_{{ATF}_{t}}$ are the proportions of prey taxa *t* (by weight) in the stomachs of Pacific Halibut (*PH*) and Arrowtooth Flounder (*ATF*) and *Q* is the total number of prey taxa observed. Estimates of dietary overlap were calculated across the uniform grid system described for spatial overlap, though grid cells containing fewer than three non-empty stomachs for each predator in a given survey year were excluded. This resulted in estimates of dietary overlap for 123 unique combinations of survey year and grid cell. Like spatial overlap, the possible range for dietary overlap estimates was between 0 (complete separation) and 1 (complete overlap). Area- and year-specific dietary overlap was quantified as described for spatial overlap.

### Resource partitioning

We used a Pearson’s correlation test to quantify the relationship between spatial and dietary overlap for Pacific Halibut and Arrowtooth Flounder in the Gulf of Alaska. Dietary overlap was calculated for fewer grid cells than spatial overlap, therefore cells containing spatial overlap estimates but not dietary overlap estimates were excluded from this analysis. We calculated correlation coefficients and p-values at the basin-wide scale, using all complementary estimates of spatial and dietary overlap. Because sampling effort was spatially variable (*i*.*e*., effort was greatest in the western and central areas of the Gulf of Alaska and lowest in the eastern region), we also tested for correlations within each INPFC statistical area and IPHC regulatory area.

## Results

### Spatial distributions

A total of 9,352 survey tows were conducted in the Gulf of Alaska between 1990 and 2017. Of these, 716 were excluded due to missing depth and bottom temperature data. Consequently, 8,636 tows were used to construct species-specific models for presence-absence (Tables 1 and 2). From this subset of tows, 59.1% (5,104) caught Pacific Halibut and 85.9% (7,422) caught Arrowtooth Flounder, and were used to construct species-specific models of CPUE. The majority (n = 422) of excluded tows were because of missing bottom temperatures from the Shumagin INPFC statistical area in 1990.

**Table 1:** Number of tows that captured at least one Pacific Halibut (n = 5,104) measuring 30 to 69 cm fork length.

INPFC Area	1990	1993	1996	1999	2001	2003	2005	2007	2009	2011	2013	2015	2017
Shumagin	5	144	124	118	111	186	142	172	169	146	115	136	104
1,672 (2,029)	(5)	(166)	(169)	(143)	(136)	(229)	(176)	(205)	(196)	(162)	(136)	(182)	(124)
Chirikof	16	98	100	79	75	107	103	138	134	109	94	133	77
1,263 (1,955)	(25)	(168)	(168)	(161)	(133)	(170)	(174)	(196)	(186)	(155)	(126)	(175)	(118)
Kodiak	32	124	68	112	86	137	146	151	145	145	114	193	115
1,568 (2,809)	(78)	(210)	(186)	(242)	(189)	(242)	(287)	(257)	(275)	(226)	(187)	(252)	(178)
Yakutat	35	46	27	53	0	26	29	21	37	25	25	37	29
390 (1,056)	(117)	(117)	(105)	(132)	(76)	(0)	(90)	(57)	(83)	(68)	(61)	(80)	(70)
Southeastern	2	4	25	15	0	22	31	20	19	22	9	25	17
211 (787)	(61)	(65)	(88)	(64)	(0)	(78)	(92)	(64)	(72)	(54)	(38)	(66)	(45)
Total	90	416	344	377	272	478	451	502	504	447	357	524	342
	(286)	(726)	(716)	(742)	(458)	(795)	(819)	(779)	(812)	(665)	(548)	(755)	(535)

Numbers are shown by International North Pacific Fisheries Commission (INPFC) statistical area and survey year. The total numbers of tows conducted are shown in parentheses (n = 8,636). Only tows with complete environmental data were tabulated.

**Table 2:** Number of tows that captured at least one Arrowtooth Flounder (n = 7,422) measuring 30 to 69 cm fork length.

INPFC Area	1990	1993	1996	1999	2001	2003	2005	2007	2009	2011	2013	2015	2017
Shumagin	4	142	148	137	103	208	170	190	180	153	116	158	114
1,823 (2,029)	(5)	(166)	(169)	(143)	(136)	(229)	(176)	(205)	(196)	(162)	(136)	(182)	(124)
Chirikof	18	144	142	142	108	139	150	152	163	137	107	144	106
1,652 (1,955)	(25)	(168)	(168)	(161)	(133)	(170)	(174)	(196)	(186)	(155)	(126)	(175)	(118)
Kodiak	62	183	167	202	157	203	258	213	241	204	162	221	166
2,439 (2,809)	(78)	(210)	(186)	(242)	(189)	(242)	(287)	(257)	(275)	(226)	(187)	(252)	(178)
Yakutat	57	98	98	115	0	70	80	50	77	64	58	78	63
908 (1,056)	(117)	(117)	(105)	(132)	(76)	(0)	(90)	(57)	(83)	(68)	(61)	(80)	(70)
Southeastern	23	27	72	50	0	64	77	53	64	45	34	54	37
600 (787)	(61)	(65)	(88)	(64)	(0)	(78)	(92)	(64)	(72)	(54)	(38)	(66)	(45)
Total	164	594	627	646	368	684	735	658	725	603	477	655	486
	(286)	(726)	(716)	(742)	(458)	(795)	(819)	(779)	(812)	(665)	(548)	(755)	(535)

Numbers are shown by International North Pacific Fisheries Commission (INPFC) statistical area and survey year. The total numbers of tows conducted (n = 8,636) are shown in parentheses. Only tows with complete environmental data were tabulated.

We identified full GAMs, which accounted for effects of survey year, tow location, depth, and bottom temperature, as the best-fit models for quantifying presence-absence of Pacific Halibut and CPUE of both Pacific Halibut and Arrowtooth Flounder (Table 3; S1 and S2 Appendices; S1 Table). Though it is commonplace and can be considered best practice to select the most parsimonious model when ΔAIC is less than two [44], we selected the full model (rather than the one that excluded bottom temperature) for presence-absence of Arrowtooth Flounder. This is because including all model covariates provided consistency for predictions across species and response types (Table 3).

**Table 3:** Results for the top three models, by species and response type (presence-absence, CPUE, where present).

	Variables Included in Alt. Model										
Model	Year	Lon, Lat	Depth	Temp	Dev. (%)		df	logLik	ΔAIC	W_i_	GCV
**Pacific Halibut**											
Presence-absence	**X**	**X**	**X**	**X**	**45.6**		**48**	**- 3179**	**0.0**	**0.997**	**- 0.253**
	X	X	X		45.4		44	- 3188	11.5	0.003	- 0.251
		X	X	X	43.9		35	- 3279	174.1	0.000	- 0.232
CPUE, where present	**X**	**X**	**X**	**X**	**46.4**		**52**	**- 7120**	**0.0**	**1.000**	**0.973**
	X	X	X		46.2		49	- 7132	17.7	0.000	0.967
		X	X	X	44.7		40	- 7199	133.5	0.000	0.999
**Arrowtooth Flounder**											
Presence-absence	X	X	X		40.1		49	- 2099	0.0	0.637	- 0.503
	**X**	**X**	**X**	**X**	**40.2**		**50**	**- 2098**	**1.1**	**0.363**	**- 0.503**
		X	X	X	33.1		38	- 2347	474.6	0.000	- 0.448
CPUE, where present	**X**	**X**	**X**	**X**	**40.3**		**53**	**- 12745**	**0.0**	**1.00**	**1.842**
	X	X	X		40.0		50	- 12764	33.4	0.00	1.850
		X	X	X	39.0		41	- 12827	140.6	0.00	1.877

X indicates the variables (survey year, longitude and latitude, depth [m], bottom temperature [°C]) included in each alternative model. The deviance explained (Dev., %), degrees of freedom (df), log likelihood (logLik), Δ AIC, Akaike weight (W_i_), and generalized cross validation (GCV) score are also noted. The selected model for each case is shown in bold.

Model results for presence-absence indicated that Pacific Halibut and Arrowtooth Flounder were commonly encountered throughout the time series (S1 Appendix). The likelihood of capturing Pacific Halibut decreased from 1990 to 2001, but generally increased thereafter. With the exception of 1990, Arrowtooth Flounder were nearly always sampled by the bottom trawl. All other variables held constant, Pacific Halibut were most often caught in the western Gulf of Alaska (Shumagin, Chirikof, and Kodiak INPFC areas; IPHC regulatory areas 4A, 3B, and the western half of 3A), at depths shallower than 100 m and in temperatures cooler than 9°C. Although Arrowtooth Flounder were observed in almost every haul (regardless of geographic location or bottom temperature), encounter rates were greatest at intermediate depths and substantially declined when tows were conducted in areas shallower than 100 m or deeper than 450 m (S1 Appendix).

Model results for CPUE (number per ha) were more variable than those for presence-absence (S2 Appendix). Though year-to-year variation in CPUE was less than one fish per hectare, Pacific Halibut CPUE generally increased from 1990 to 2017, whereas Arrowtooth Flounder CPUE generally decreased (S2 Appendix). CPUE for Pacific Halibut was greatest near Unimak Pass (Shumagin area, IPHC regulatory area 4A) and along the continental shelf-slope break in the eastern region. Arrowtooth Flounder CPUE was greatest in Shelikof Strait (located between Kodiak Island and the Alaska Peninsula) and south to Unimak Island (Shumagin, Chirikof, and Kodiak INPFC areas; IPHC regulatory areas 4A, 3B, and the western half of 3A). Pacific Halibut CPUE peaked at approximately 50 m depth and Arrowtooth Flounder CPUE peaked near 150 m and 350 m. Both species displayed steep declines in CPUE on either side of their respective mode(s). Finally, Pacific Halibut CPUE remained relatively high in waters colder than 9°C, whereas Arrowtooth CPUE increased with increasing bottom temperature (S2 Appendix).

**Fig 3:**
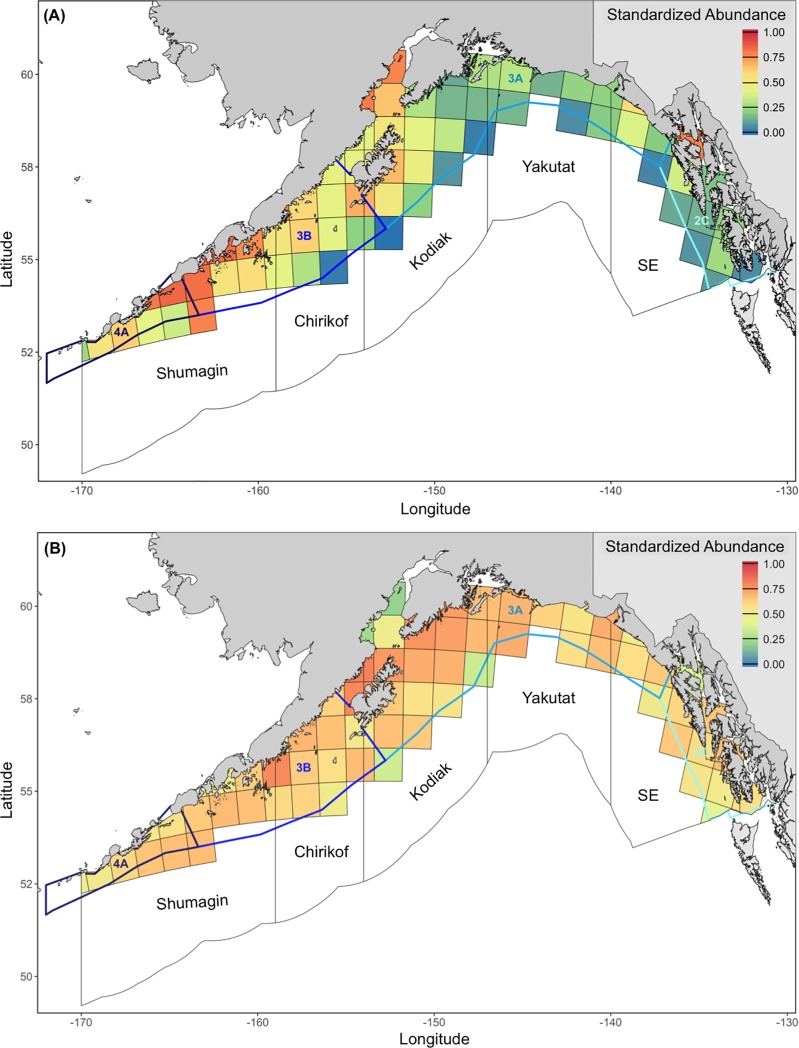
**Mean standardized abundances for (A) Pacific Halibut and (B) Arrowtooth Flounder (1990 to 2017).** Filled squares represent individual 100 km x 100 km grid cell estimates. Polygons denote International North Pacific Fisheries Commission (INPFC) statistical areas (black outlines) and International Pacific Halibut Commission (IPHC) regulatory areas (blue-shaded outlines) in the Gulf of Alaska.

Standardized abundance estimates, which combined the probability of occurrence and predicted abundance at a particular time and place, displayed distinct spatial patterns for Pacific Halibut and Arrowtooth Flounder. Standardized abundances for Pacific Halibut were greatest in Cook Inlet, along the east coast of Kodiak Island, and near Unimak Pass, lowest in the central Gulf of Alaska (Kodiak and Yakutat INPFC areas and IPHC area 3A), and moderate to low in the eastern region (Southeastern INPFC area and IPHC area 2C) (Fig 3A). Except for Cook Inlet and the deepest areas of the Gulf of Alaska (*i*.*e*., the continental shelf-slope break), standardized abundances for Arrowtooth Flounder were relatively high (Fig 3B). Grid cell-specific probabilities of occurrence, predicted abundances, and standardized abundance estimates did not vary considerably by survey year.

### Spatial overlap

**Fig 4:**
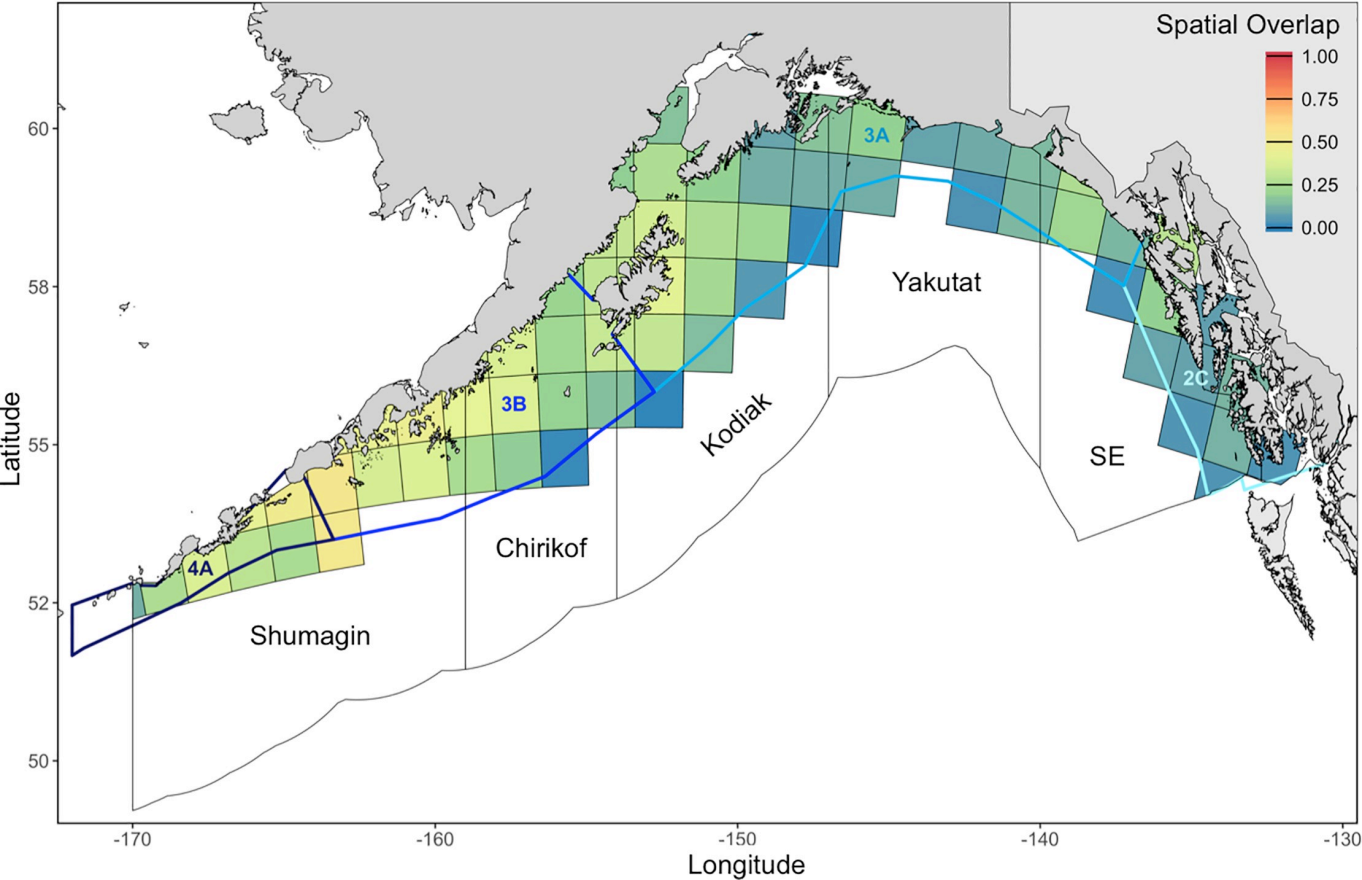
Mean spatial overlap between Pacific Halibut and Arrowtooth Flounder (1990 to 2017). Filled squares represent individual 100 km x 100 km grid cell estimates. Polygons denote International North Pacific Fisheries Commission (INPFC) statistical areas (black outlines) and International Pacific Halibut Commission (IPHC) regulatory areas (blue-shaded outlines) in the Gulf of Alaska.

Overall patterns in spatial overlap (Fig 4) between Pacific Halibut and Arrowtooth Flounder closely resembled patterns in Pacific Halibut abundance. Though means ranged from 0.00 (no overlap) to 0.61 (moderate to high overlap) at the survey year-grid cell level, Pacific Halibut and Arrowtooth Flounder exhibited low spatial overlap (0.26 ± 0.13 SD) at the basin-wide scale. ANCOVA results indicted no significant interaction between survey year and INPFC or IPHC area (INPFC F_47,799_ = 0.34 p > 0.99; IPHC F_34,768_ = 0.38, p > 0.99). There were, however, main effects of year (INPFC F_12,846_ = 11.46, p < 0.001; IPHC F_12,802_ = 9.26, p < 0.01) and area (INPFC F_4,846_ = 152.94, p < 0.001; IPHC F_3,802_ = 145.99, p < 0.001) on spatial overlap. Mean spatial overlap slightly increased throughout the time series and from east to west. Substantial overlap was found along the northeast side of Kodiak Island, the western half of the Alaska Peninsula, and near Unimak Pass. Grid cells with the greatest spatial overlap (S ≥ 0.60; n = 2) measured 31 to 112 m depth and 2.7 to 7.2°C.

The Tukey HSD test revealed differences in spatial overlap among all area-level combinations except between the Yakutat and Southeastern INPFC statistical areas and between IPHC regulatory areas 4A and 3B. On average, spatial overlap was highest in the Shumagin INPFC statistical area (0.36 ± 0.13 SD) and IPHC regulatory areas 4A and 3B (0.33 ± 0.14 SD) (Fig 4). These overlap estimates were followed by Chirikof (0.26 ± 0.12 SD) and Kodiak (0.21 ± 0.11 SD) INPFC statistical areas and IPHC regulatory area 3A (0.19 ± 0.11 SD). The lowest estimate of spatial overlap was found in the eastern Gulf of Alaska (*i*.*e*., Yakutat and Southeastern INPFC statistical areas: 0.13 ± 0.08 SD; IPHC regulatory area 2C: 0.10 ± 0.07 SD).

### Diet compositions

**Fig 5:**
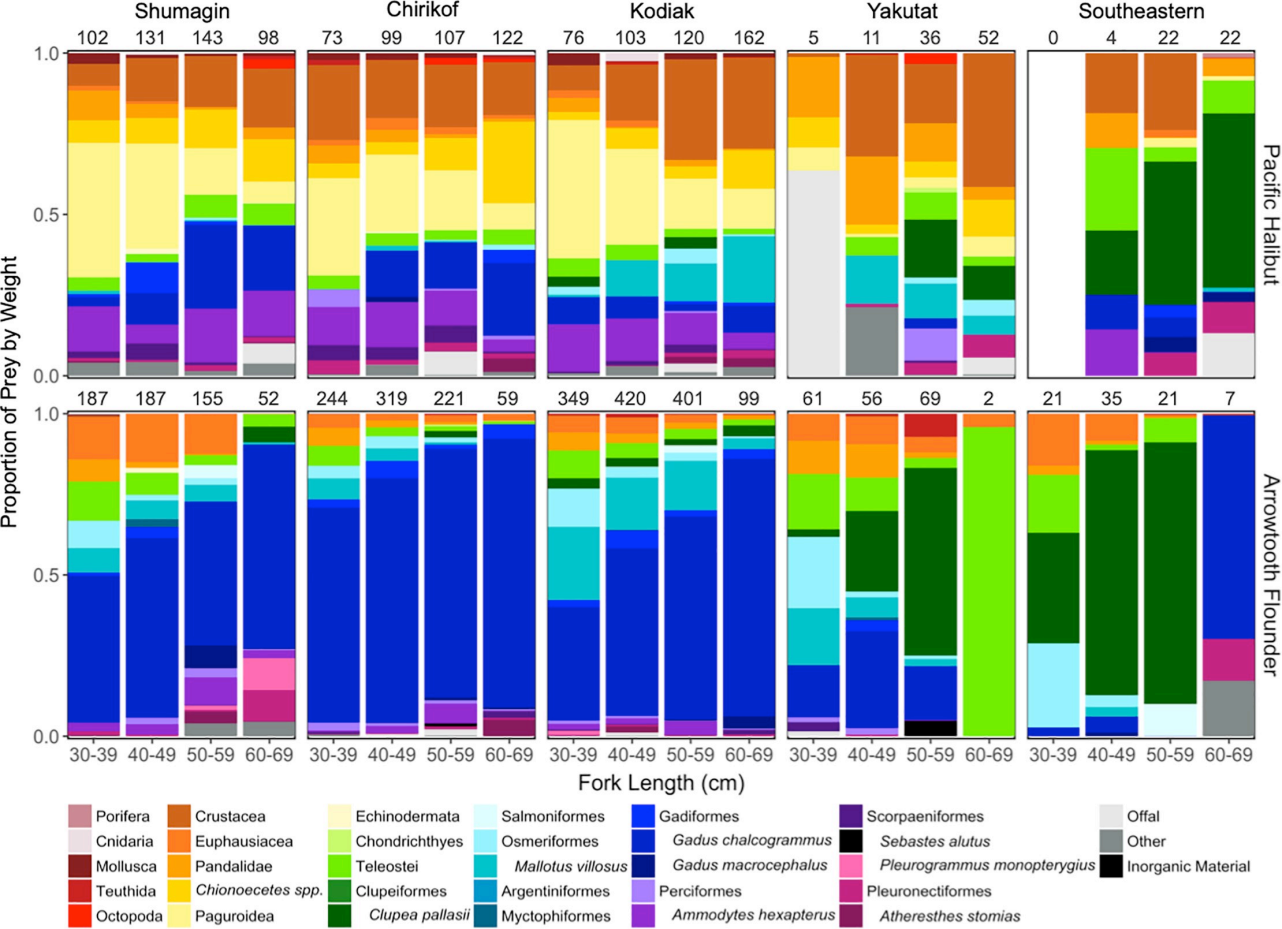
Mean proportions of prey by weight for Pacific Halibut and Arrowtooth Flounder (all survey stations, 1990 to 2013). Calculations were grouped by International North Pacific Fisheries Commission (INPFC) statistical area and size class. Sample sizes are indicated above each stacked bar. Prey taxa that constituted less than 0.01 by weight were classified into broader taxonomic groups (*e*.*g*., phyla for invertebrate taxa and order for fishes).

Pacific Halibut and Arrowtooth Flounder consumed similar species of prey, though in different proportions. Subsampling for gut content analysis resulted in 1,881 Pacific Halibut stomachs and 5,163 Arrowtooth Flounder stomachs. Of these, 1,488 Pacific Halibut and 2,965 Arrowtooth Flounder contained one or more prey items (Table 4). Approximations of stomach fullness for those sampled with contents indicated that 44.4% of Pacific Halibut and 43.0% of Arrowtooth Flounder stomachs were between half full and distended. When combining all years and areas, both Pacific Halibut and Arrowtooth Flounder consumed 59 different prey taxa. Of these, 47 were common to both predators. Invertebrates and fishes constituted approximately equal proportions by weight of Pacific Halibut diets (fishes = 0.57, invertebrates = 0.43), whereas fishes dominated the diets of Arrowtooth Flounder (fishes = 0.93, invertebrates = 0.07). Generally, Pacific Halibut diets were more diverse (H’ = 2.72) and even (J’ = 0.58) than diets of Arrowtooth Flounder (H’ = 1.72, J’ = 0.37; S2 Fig). This was due to the wide variety of invertebrate prey consumed by Pacific Halibut (*e*.*g*., crabs and shrimps (49.9%), cephalopods (1.1%), and other benthic invertebrates (1.2%)). Proportions of prey by weight varied by area and size class for both Pacific Halibut and Arrowtooth Flounder (Fig 5). For instance, invertebrates were consumed in greater proportions by smaller fish and Pacific Herring (*Clupea pallasii*) made up relatively large proportions of the diets for both predators, but only in the eastern Gulf of Alaska.

**Table 4:** Number of non-empty stomachs sampled, by International North Pacific Fishery Commission (INPFC) statistical area and survey year.

INPFC Area	1990	1993		1996	1999	2001	2003	2005	2007	2009	2011	2013	Total
Shumagin	15		50	31	3	98	26	13	55	37	43	103	474
	18		21	138	25	85	49	11	44	55	56	79	581
Chirikof	28		42	22	0	79	31	7	16	44	36	96	401
	106		87	219	58	135	44	16	35	43	43	57	843
Kodiak	39		22	30	37	75	9	1	58	40	48	102	461
	143		111	244	94	280	42	26	34	74	97	124	1,269
Yakutat	2		9	0	0	0	3	7	17	14	17	35	104
	14		28	1	0	0	10	15	19	39	23	39	188
Southeastern	0		0	0	0	0	0	4	11	10	7	16	48
	0		0	0	0	0	5	12	15	25	13	14	84
Total	84		123	83	40	252	69	32	157	145	151	352	1,488
	281		247	602	177	500	150	80	147	236	232	313	2,965

Numbers for Pacific Halibut are listed as the top line in each category and Arrowtooth Flounder are shown below. Food habits data were not yet available for 2015 or 2017.

### Dietary overlap

**Fig 6:**
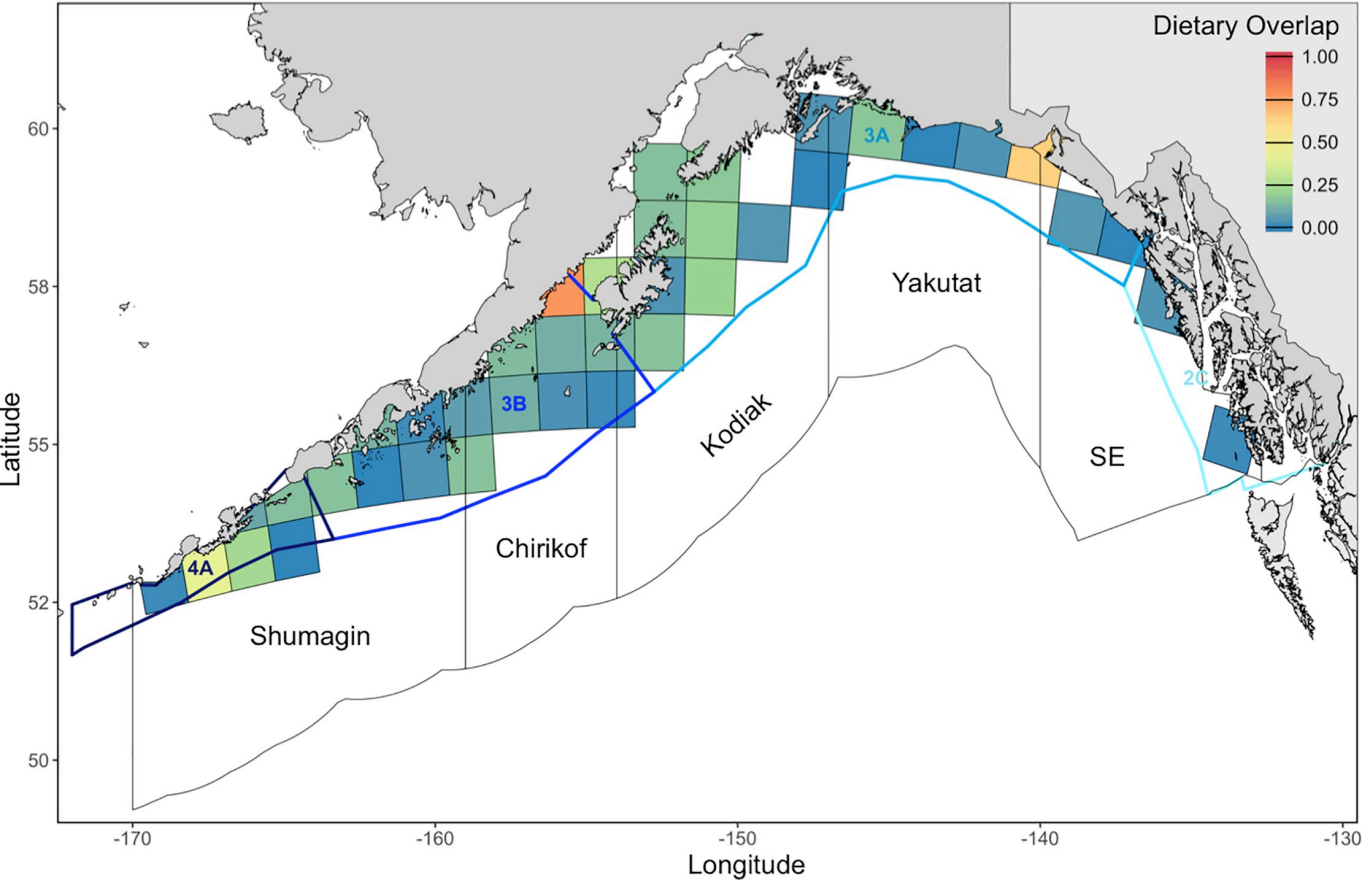
Mean dietary overlap between Pacific Halibut and Arrowtooth Flounder (1990 to 2013). Filled squares represent individual 100 km x 100 km grid cell estimates. Polygons denote International North Pacific Fisheries Commission (INPFC) statistical areas (black outlines) and International Pacific Halibut Commission (IPHC) regulatory areas (blue-shaded outlines) in the Gulf of Alaska.

Estimates of dietary overlap ranged from 0.00 (no overlap) to 0.81 (high overlap) at the survey year-grid cell level, but the basin-wide mean was considerably low (0.13 ± 0.20 SD; Fig 6). We found no significant interactions between survey year and area (ANCOVA: INPFC F_23,116_ = 0.42, p = 0.99; IPHC F_17,120_ = 0.35, p > 0.99). There was also no main effect of area on dietary overlap (INPFC F_4,139_ = 0.59, p = 0.67; IPHC F_3,137_ = 1.11, p = 0.35). There were, however, differences in dietary overlap with survey year (INPFC: F_10,139_ = 2.14, p = 0.03, IPHC: F_10,137_ = 2.57, p > 0.01). Grid cells with the greatest dietary overlap (D > 0.60; n = 9) measured 134 ± 44 m depth and 5.6 ± 1.2°C.

### Resource partitioning

**Fig 7:**
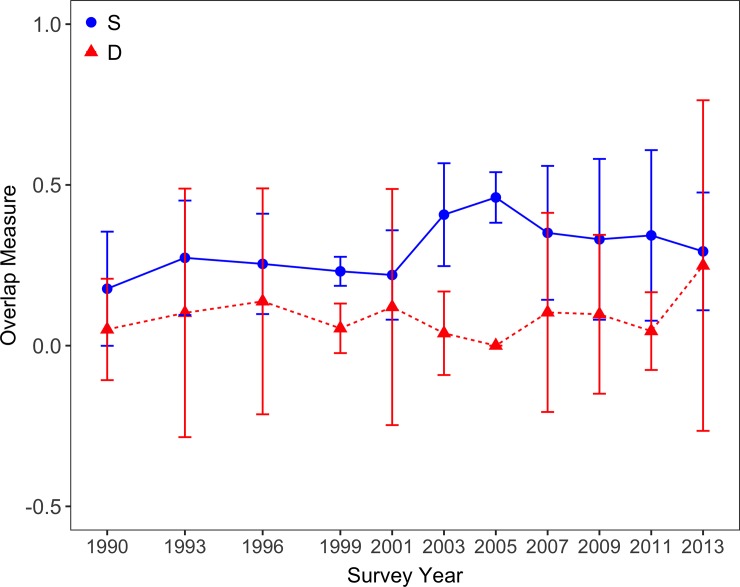
Niche overlap estimates for Pacific Halibut and Arrowtooth Flounder in the Gulf of Alaska (1990 to 2013). Spatial overlap (S) is denoted by blue circles and solid lines. Dietary overlap (D) is denoted by red triangles and dashed lines.

Pearson’s correlation tests revealed no significant relationship between spatial overlap and dietary overlap for Pacific Halibut and Arrowtooth Flounder in the Gulf of Alaska (Fig 7; S3 Fig). This was true at the basin-wide scale (r_108_ = - 0.02, t_130_ = - 0.20, p = 0.84) and when areas were tested separately (correlation coefficients for INPFC areas ranged from– 0.02 in Kodiak to 0.37 in Southeastern and correlation coefficients for IPHC areas ranged from– 0.06 in 3B to 0.72 in 2C; all p-values > 0.1).

## Discussion

Pacific Halibut and Arrowtooth Flounder distributions and abundances varied as a function of survey year, location (*i*.*e*., latitude and longitude), depth, and bottom temperature. However, year, location, and temperature had much less of an effect on shaping Arrowtooth Flounder distributions. Given the ubiquity of Arrowtooth Flounder in the Gulf of Alaska, we found that patterns in spatial overlap were largely driven by the distributions and abundances of Pacific Halibut. We found support for the hypothesis that spatial overlap between Pacific Halibut and Arrowtooth Flounder would be greatest at intermediate depths (73 to 90 m) and temperatures (2.7 to 8.3°C). Contrary to our expectations, spatial overlap did not vary substantially by year and was not at its greatest during periods of high halibut spawning stock biomass. Diet compositions were most similar for the larger (*i*.*e*., 30 to 69 cm) size classes analyzed in this study, as anticipated. Sparse stomach sampling in both time and space led to relatively few unique combinations of survey year and grid cell, thus we were unable to make strong inferences about spatiotemporal patterns in dietary overlap. Estimates of dietary overlap were generally low throughout the study area, despite our hypothesis that low species diversity in the western Gulf of Alaska would lead to greater overlap in the diets of Pacific Halibut and Arrowtooth Flounder. Finally, resource partitioning between Pacific Halibut and Arrowtooth Flounder was not apparent in the Gulf of Alaska given that there was no correlation between spatial overlap and dietary overlap at the scale of our analyses.

### Spatial distributions and spatial overlap

Pacific Halibut were most often encountered in the relatively cold (< 5°C), shallow (< 100 m) waters of the western Gulf of Alaska. Observed distributions for the size range of halibut assessed reflect known movement patterns, with smaller individuals more frequently occupying the western Gulf of Alaska before emigrating eastward [58]. However, relatively high densities of Pacific Halibut in the western Gulf of Alaska may also be confounded with the survey design, which consistently moves from west to east as the summer progresses [29]. This is because temporary aggregations of prey may be found in the western Gulf of Alaska at the time of data collection due to localized increases in primary productivity in late spring [59].

Arrowtooth Flounder were observed in virtually every survey year and tow location, demonstrating a wide spatial niche breadth in the Gulf of Alaska. The greatest catch rates for Arrowtooth Flounder were in moderately deep (200 to 300 m) waters of Shelikof Strait. Though Arrowtooth Flounder were encountered in similar frequencies regardless of temperature, CPUE increased in warmer waters. The relationship between Arrowtooth Flounder abundance and temperature is corroborated by observations made in the Eastern Bering Sea, where Arrowtooth Flounder prefer warmer waters and actively avoid the “cold pool” (*i*.*e*., temperatures < 2°C) [23,60].

Because Arrowtooth Flounder were so ubiquitous throughout the Gulf of Alaska, patterns in spatial overlap were primarily driven by distributions of Pacific Halibut. The only major exception was in the shallow (< 100 m) waters of Cook Inlet, where relatively few Arrowtooth Flounder were found. The western Gulf of Alaska, which is characterized by a broader continental shelf, greater amounts of shallow water (< 200 m) habitat, and colder (< 5°C) bottom temperatures resulted in higher spatial overlap than the eastern Gulf of Alaska, which is characterized by a relatively narrow continental shelf and warmer (> 5°C) bottom temperatures. Additionally, moderate to high estimates of spatial overlap may be attributable to the greater productivity, higher groundfish densities, and lower overall species diversity in the western Gulf of Alaska [21,59]. At the finer grid cell level, there was a wide range of spatial overlap values with few high estimates suggesting more localized species-specific responses to exogenous factors. However, we cannot distinguish whether estimates of spatial overlap result from competitive interactions or some other variable (*e*.*g*., habitat suitability, prey availability) using only species’ distributions and abundances. Therefore, we evaluated the linear relationship between spatial overlap and dietary overlap to provide insight into the role of competition as a plausible driver of observed patterns of resource partitioning.

### Diet compositions and dietary overlap

We found that diet compositions of Pacific Halibut were more diverse and benthically associated than Arrowtooth Flounder. This is comparable to findings from previous studies, which have shown a) wider varieties of fish and invertebrates consumed by Pacific Halibut and b) that crabs constitute greater proportions of prey by weight in diets of small Pacific Halibut, whereas Walleye Pollock (*Gadus chalcogrammus*) dominate the diets of similarly sized Arrowtooth Flounder [11,24–25,27,61]. Diet compositions of Pacific Halibut and Arrowtooth Flounder were more similar at larger sizes due to the greater proportions of fish consumed by Pacific Halibut. Although a relatively wide dietary niche likely provides Pacific Halibut with greater flexibility in responding to fluctuating community compositions and nearby competitors [20], prey switching may have metabolic consequences (*e*.*g*., decreased growth [11]). This is especially true if that shift is directed from higher quality, energy dense prey to lower quality taxa, as inferred from differences in diets between fast- and slow-growing halibut [11–12]. However, interpreting changes in diet compositions is context-dependent and requires information about prey availability and predator preferences.

Given differences in diet compositions, we found dietary overlap between Pacific Halibut and Arrowtooth Flounder to be low, but highly variable throughout the Gulf of Alaska. Mean dietary overlap was greatest in a single grid cell in the eastern Gulf of Alaska, where more Pacific Herring (*Clupea pallasii*) were consumed by both species. The increased proportions of herring in the diets of Pacific Halibut and Arrowtooth Flounder sampled from Southeast Alaska, specifically in 2005, coincided with relatively high herring biomass during late summer [62]. This particular grid cell is also located in close proximity to a herring spawning stock boundary designated by the Alaska Department of Fish and Game [62]. Feeding on locally abundant prey is evidence of the opportunistic nature of these predators, which likely exhibit prey switching in response to prey populations.

A necessary caveat when comparing the diets of Pacific Halibut and Arrowtooth Flounder is that stomach fullness and diet compositions may, in part, reflect differential responses to capture and handling. Arrowtooth Flounder are relatively soft-bodied fish that tend to regurgitate more frequently when disturbed [31] (Barnes pers. obs). This physiological stress response could be responsible for the greater proportion (approximately 2.5 times) of empty stomachs for Arrowtooth Flounder when compared to Pacific Halibut [24]. Though fish displaying signs of regurgitation were excluded as part of the sampling protocol [26], it is difficult to know for certain whether or not partial regurgitation occurred before fish made it to the sampling table. Additionally, discarding fish suspected of regurgitation could bias sampling toward fish with partially full or empty stomachs [31] and away from those feeding most heavily, as fish with the fullest stomachs may be prone to regurgitation.

### Resource partitioning and the potential for competition

Niche-based competition theory states that the coexistence of competing species is only possible through resource partitioning, which differentiates the ecological requirements of species to prevent competitive exclusion [16, 20, 63]. Despite opposing trajectories of Pacific Halibut growth and Arrowtooth Flounder biomass at the basin-wide scale [13], we did not detect resource partitioning (*i*.*e*., a negative relationship between spatial overlap and dietary overlap) as would be expected if competition was ongoing or had taken place in the recent past. With low (eastern Gulf of Alaska) to moderate (western and central Gulf of Alaska) overlap in space and generally low overlap in diet, it is possible that the two species require different enough resources to preclude competition (*i*.*e*., overlap estimates reflect the virtual niche of each species rather than actual niche breadths that had been constricted due to competition). If this were the case, bottom-up processes would be responsible for variation in capture probability, relative abundance, diet composition, and niche overlap. For example, the niche breadth of Pacific Halibut might be restricted to shallower waters, cooler temperatures, and invertebrate prey regardless of whether or not Arrowtooth Flounder occupy deeper depths and warmer waters or more heavily rely on fish as prey (or vice versa; S1 and S2 Appendices). Given historically low size-at-age of Pacific Halibut [4], it is also plausible that disparate responses to environmental change (*e*.*g*., recruitment) are responsible for recent changes in the population trajectories of Pacific Halibut (decreasing) and Arrowtooth Flounder (increasing) [14]. Notably, however, a lack of evidence for resource partitioning may also be due to a divergence in resource use prior to the collection of necessary data. Though fishery catch data and various survey data are available prior to the most recent declines in Pacific Halibut size-at-age (*e*.*g*. [4,8]), a lack of standardized methods and sparse diet information prevent an analysis of resource partitioning before 1990.

Several factors could have impacted our ability to assess resource partitioning between Pacific Halibut and Arrowtooth Flounder. One reason we may have been unable to detect a relationship between spatial and dietary overlap is a low signal to noise ratio. When tracking paired means for spatial and dietary overlap through time, there appeared to be a negative correlation, especially from 2001 onward (Fig 7). With a few exceptions, where there was no change in spatial overlap from one survey year to the next, an increase in the mean overlap along one dimension corresponded with a decrease in the mean overlap for the other. However, confidence intervals indicated that 1999, 2003, and 2005 were the only survey years to yield statistically distinct estimates of spatial and dietary overlap, making the detection of any pattern (should one exist) impossible at the basin-wide scale. This low signal to noise ratio persisted at finer (*i*.*e*., INPFC and IPHC area) spatial scales, though the degree of habitat heterogeneity encompassed by statistical or regulatory areas likely continues to mask interactions at this scale. Sample size limitations and a need to aggregate diet data precluded an assessment of patterns in resource use at scales finer than the uniform 100 km x 100 km grid cell, which still may be too broad to detect ecologically relevant interactions between the two species.

Effectively characterizing the resource use of marine fishes, especially those with opportunistic foraging strategies, requires a large number of samples (ideally ≥ 50 observations per grouping) [64]. Low sample sizes generally increase variation in diet compositions, make it difficult to detect patterns in consumption of prey, and can result in underestimations of niche overlap [65]. Interestingly, the number of moderate to high estimates of dietary overlap (D ≥ 0.40) appeared to increase with sample size. The spatiotemporal coverage of dietary overlap estimates was sparse in part because we required at least three non-empty Pacific Halibut and Arrowtooth Flounder stomachs in each combination of survey year and grid cell. Robust estimates of diet composition are especially important, given that trophic separation is more common than spatial separation in marine systems [18,66]. As such, increased sampling for gut content analysis would enhance our understanding about the relationships between spatial and dietary overlap and whether or not competition can serve as a mechanism for changes in size-at-age. Specifically increasing sampling efforts for the Yakutat and Southeastern INPFC areas (IPHC areas 3A and 2C) and more consistent sampling through time should increase the power to detect relationships in niche overlap within the Gulf of Alaska, should they exist. However, more robust estimates of diet composition and higher spatiotemporal resolution of the dietary overlap measure may fail to improve inferences of competition under the theory of resource partitioning. This is because prey that are relatively rare in the diets of Arrowtooth Flounder could undergo local depletion as a result of high Arrowtooth Flounder abundance. If that particular prey taxon is important for Pacific Halibut, intense competitive pressure may persist even in cases of low dietary overlap, regardless of the degree of spatial overlap between the two predators.

The apparent lack of resource partitioning may have also been an artifact of selecting specific size classes of individuals for comparison purposes. We selected similar fork lengths as our basis for comparison across species because body size has been identified as more important than phylogeny in determining functional roles within a particular food web [67]. Larger individuals are often considered superior competitors because of their increased visual acuity, faster swimming speeds, and more aggressive behaviors [68]. However, smaller species can have negative effects on larger species by consuming shared prey at one or more life stage. A tractable example of these interactions has been described for the small but abundant Redside Shiner (*Richardsonius balteatus*) and relatively large Rainbow Trout (*Salmo gairdneri*) in British Columbia (*e*.*g*., [69–71]). Due to their increased population sizes and widespread distributions, shiners were able to overgraze amphipods before they attained sizes available to juvenile trout. This resulted in a need for juvenile trout to feed on suboptimal prey, thereby reducing their growth rates at early stages. Based on the differences in diet compositions of Pacific Halibut and Arrowtooth Flounder and rates at which each predator becomes increasingly piscivorous, it is possible that some other combination of sizes (*e*.*g*., 20 to 29 cm Arrowtooth Flounder and 60 to 69 cm Pacific Halibut) is more appropriate to assess resource partitioning and infer competition between the two species. In fact, Yang [24] found that dietary overlap was highest for Arrowtooth Flounder ≥ 40 cm and Pacific Halibut ≥ 80 cm. However, we lacked sufficient data to restrict size ranges in this way.

Finally, we may have been unable to detect resource partitioning because of numerous, interacting drivers of halibut size-at-age. If competition between Pacific Halibut and Arrowtooth Flounder was at least in part responsible for declines in halibut size-at-age, its effects could have been moderated by various impacts from environmental variation [11,31,72] or masked by other ecological interactions such intensified intraspecific competition during periods of high Pacific Halibut biomass *e*.*g*., ([73]). Consequences of size-selective fishing (as identified by Sullivan [13]) within the size ranges analyzed for this study, however, should be minimal. In short, we have necessarily used simple models with a set of *a priori* assumptions to study one component of a highly complex ecological system, thereby increasing the likelihood of interpretive error [74]. Additionally, any identified mechanism would not explain an observed pattern at all spatial or temporal scales [75–76]. Continued data collection would enhance our understanding about changing niche requirements of Pacific Halibut and Arrowtooth Flounder and how interactions between the two species may vary in time, space, and under different environmental conditions. Standardized surveys that focused on a few dominant groundfish prey (*e*.*g*., various crabs, pollock, and herring) would also provide context for interpreting spatiotemporal changes in niche overlap [17,77–78]. It would be valuable to collect age information pertaining to fish subsampled for gut content analysis. At present, randomly-sampled fish are used to estimate age compositions of the catch from bottom trawl surveys and these ages are not linked to individual stomach samples. If these data were available, spatial models and diet analyses could be stratified by age in addition to length. Age information would also enable direct associations between diet compositions and size-at-age, which would be especially useful for future studies similar to this one. The collection of new data will be especially useful in the near-term, given a recent stabilization of Pacific Halibut size-at-age [3] and considerable reductions in Arrowtooth Flounder biomass [8].

### Implications for fisheries management

Our results are limited to the time frame of data collection, areas sampled, and sizes of included in analyses. As such, conclusions presented herein can only be applied to resource use by Pacific Halibut and Arrowtooth Flounder measuring 30 to 69 cm in the Gulf of Alaska since 1990. Data were unavailable to assess resource use in non-summer months or simultaneously in all areas throughout the summer. Due to ontogenetic shifts and known seasonal migrations, spatial distributions and diet compositions are likely different for other size and age classes of fish as well as in different seasons (*i*.*e*., fall, winter, or spring). Despite these limitations, this study represents a first step toward evaluating the hypothesis that intensified competition with an increasing Arrowtooth Flounder population has contributed to decreases in mean size-at-age of Pacific Halibut in the Gulf of Alaska.

Changing community compositions is not unique to the Gulf of Alaska and spatiotemporal variation in life history is not unique to Pacific Halibut. There have been increases in the frequency of “native invasions” and “biotic homogenization” resulting from new niche opportunities associated with climate change [79–80]. Additionally, a number of other species (*e*.*g*., Pacific salmon, *Oncorhynchus* spp. [81–83]) have experienced changes in size-at-age, suggesting an effect of shared environmental drivers on fish growth. Given such variations in size-at-age, there is considerable value in understanding how shifts in the abundance of one species may impact life history traits of other species that are connected through their use of space or position in the food web.

We found regional patterns in spatial overlap for Pacific Halibut and Arrowtooth Flounder, with higher overlap in the western Gulf of Alaska and lower overlap in the eastern Gulf of Alaska. Declines in halibut size-at-age were also greatest in the western Gulf of Alaska when compared to lower, though highly variable, declines in the east [13,84]. A number of other studies have suggested west-east patterns in the Gulf of Alaska. Holsman *et al*. [11] found increased metabolic demands and increased foraging rates for juvenile halibut in the western and central Gulf of Alaska. More generally, Mueter and Norcross [21] found that the western Gulf of Alaska displayed greater groundfish abundances, but lower species richness and diversity than the eastern Gulf of Alaska. These clear differences in community compositions and physiological processes between east and west provide support for the spatially-explicit assessment models currently in development for Pacific Halibut, Arrowtooth Flounder, and other groundfish predators in the Gulf of Alaska (*e*.*g*., [85–86]). Incorporating spatial structure into stock assessments and fishery management plans will likely enhance our understanding about the ecological mechanisms responsible for changes in population abundance (*e*.*g*., localized adaptation, ontogenetic changes in habitat use, trophic interactions, density-dependent effects, structural changes related to fishing) [43]. It will also help us understand how components of a particular community respond to environmental cues (*e*.*g*., temperature and salinity), enabling better predictions of ecological change [87]. More broadly, results from this study improve our understanding about complex ecological interactions among economically important groundfish species at various scales and contribute to our existing knowledge about how these interactions may change in time, space, and under different environmental conditions.

## Supporting information

S1 Appendix **Partial effects of model covariates on presence (1) or absence (0) of Pacific Halibut (left) and Arrowtooth Flounder (right) in the Gulf of Alaska (1990 to 2017)**. Plots were produced using ‘visreg’ [88] and ‘mgcv’ [38] functions in R. Red lines illustrate predicted relationships from generalized additive models (GAMs) and gray bands denote 95% confidence intervals. Numbers above or below survey years denote sample sizes (*i*.*e*., the number of hauls conducted). Effective degrees of freedom (EDF) and individual data points (black ticks along x-axis) are shown for smoothed univariate covariates.
(PDF)

S2 Appendix **Partial effects of model covariates on catch-per-unit-effort (CPUE; number per ha) for Pacific Halibut (left) and Arrowtooth Flounder (right) in the Gulf of Alaska (1990 to 2017)**. Plots were produced using ‘visreg’ [88] and ‘mgcv’ [38] packages in R. Red lines illustrate predicted relationships from generalized additive models (GAMs) and gray bands denote 95% confidence intervals. Numbers above or below survey years denote sample sizes (*i*.*e*., the number of hauls conducted). Effective degrees of freedom (EDF) and individual data points (black ticks along x-axis) are shown for smoothed univariate covariates.
(PDF)

S1 Table Parameter estimates from selected generalized additive models for Pacific Halibut (A) and Arrowtooth Flounder (B). Because year was treated as a factor, 1990 is denoted as the model intercept and estimates for subsequent years are shown as differences from 1990. Smoothed variables include location (latitude, longitude), depth, and bottom temperature. Non-significant terms (α = 0.1) are grayed out.
(PDF)

S1 Fig Mean grid cell-specific estimates of abundance (1990 to 2017) for Pacific Halibut (left) and Arrowtooth Flounder (right) using different standardization methods (*i*.*e*., dividing individual grid cell abundances by the species-specific maximum, mean, or median predicted abundance).
(PDF)

S2 Fig Rarefaction curves illustrating changes in number of cumulative prey taxa encountered with sample size. Pacific Halibut is shown in red and Arrowtooth Flounder is shown in blue. Shaded areas indicate 95% confidence intervals.
(PDF)

S3 Fig Relationship between spatial overlap and dietary overlap for Pacific Halibut and Arrowtooth Flounder in the Gulf of Alaska (1990 to 2013). Each data point represents a unique combination of survey year and grid cell.
(PDF)
