# Supplementary figures and images for: Assessing the potential for competition between Pacific Halibut (Hippoglossus stenolepis) and Arrowtooth Flounder (Atheresthes stomias) in the Gulf of Alaska
Source: PLoS One. 2018 Dec 18;13(12):e0209402. doi: 10.1371/journal.pone.0209402 (PMC6298734; doi:10.1371/journal.pone.0209402)

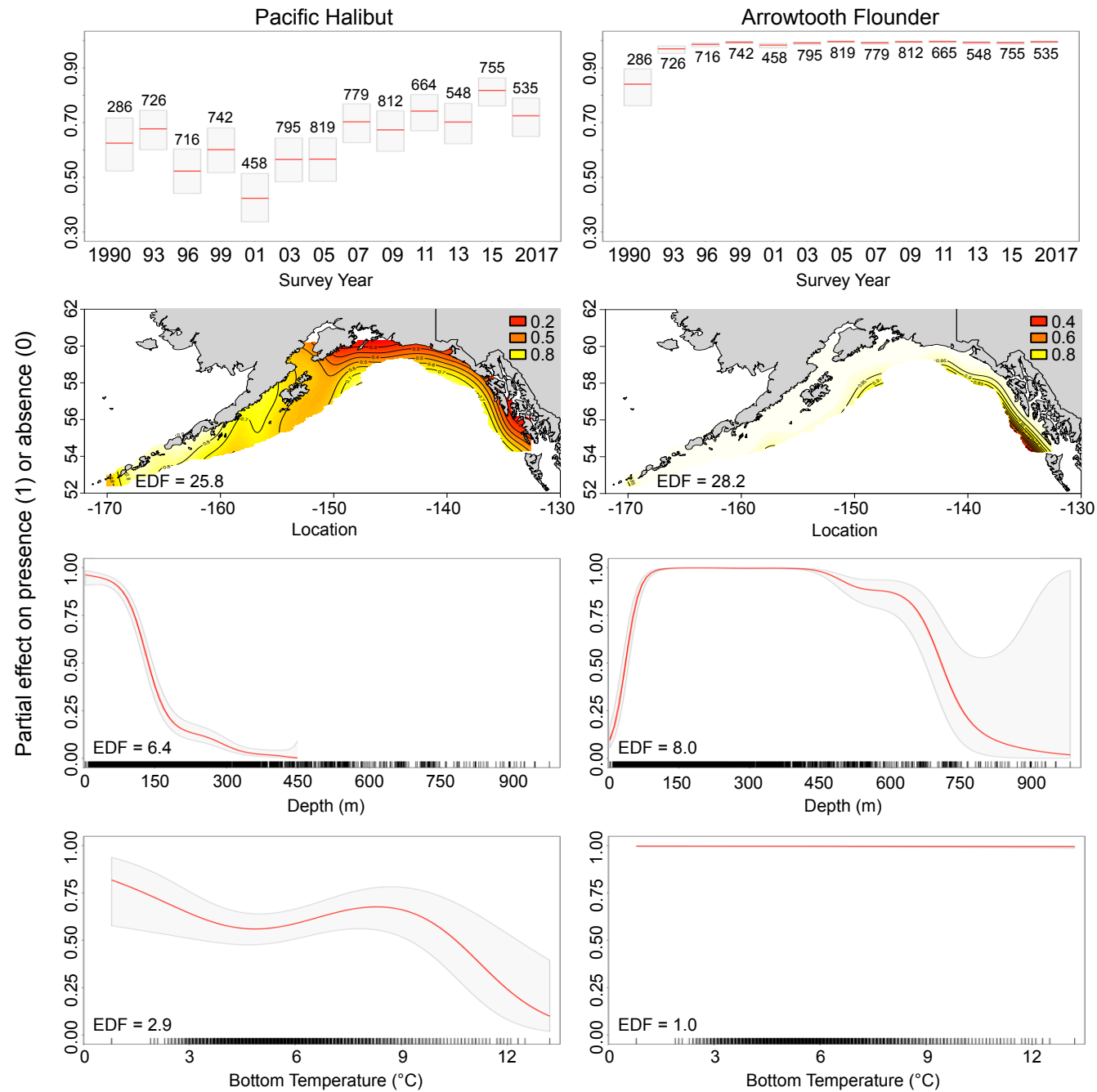

Supplement: S1 Appendix — Partial effects of model covariates on presence (1) or absence (0) of Pacific Halibut (left) and Arrowtooth Flounder (right) in the Gulf of Alaska (1990 to 2017). Plots were produced using ‘visreg’ [88] and ‘mgcv’ [38] functions in R. Red lines illustrate predicted relationships from generalized additive models (GAMs) and gray bands denote 95% confidence intervals. Numbers above or below survey years denote sample sizes (i.e., the number of hauls conducted). Effective degrees of freedom (EDF) and individual data points (black ticks along x-axis) are shown for smoothed univariate covariates. (PDF) [file pone.0209402.s001.pdf]

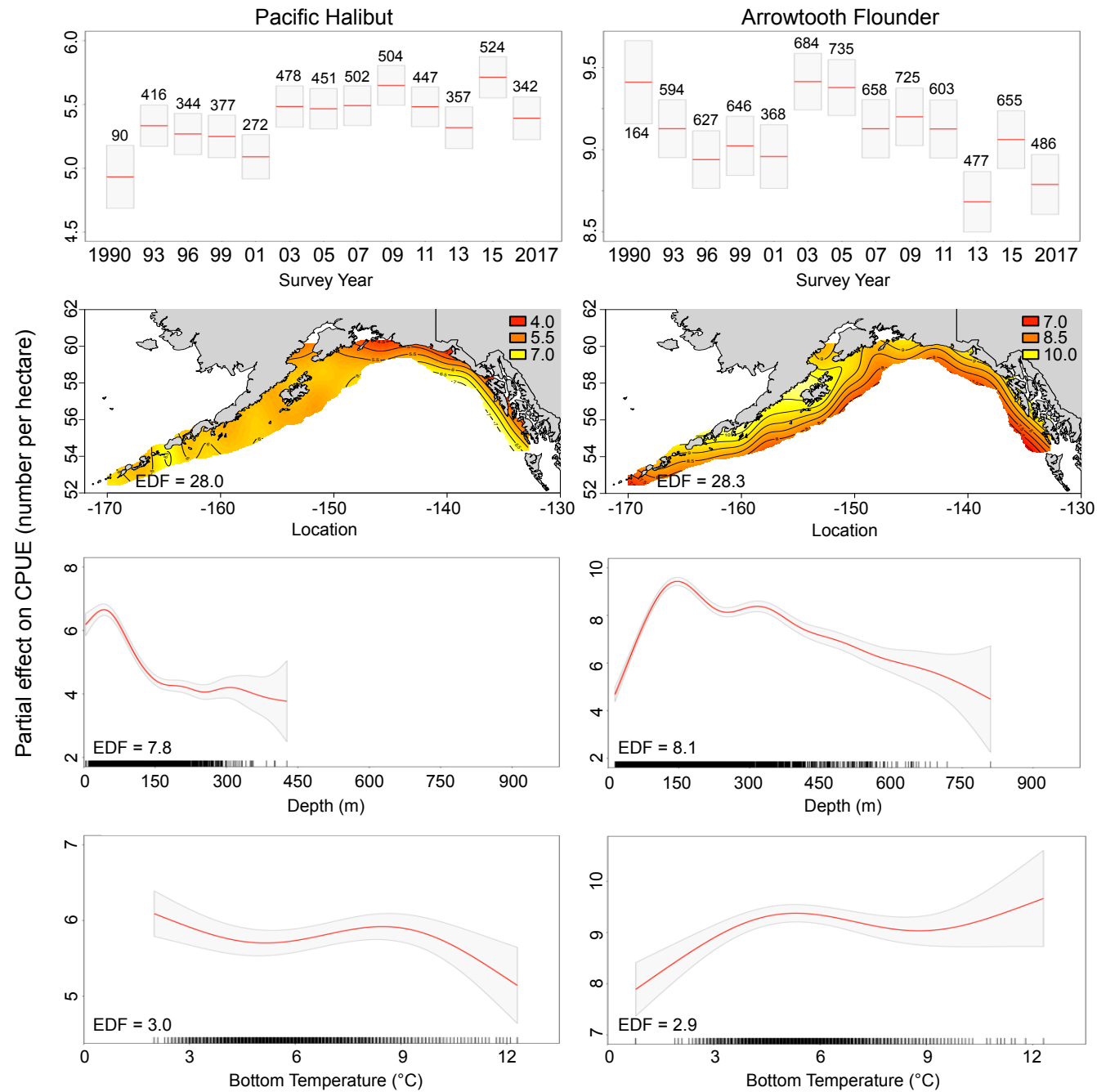

Supplement: S2 Appendix — Partial effects of model covariates on catch-per-unit-effort (CPUE; number per ha) for Pacific Halibut (left) and Arrowtooth Flounder (right) in the Gulf of Alaska (1990 to 2017). Plots were produced using ‘visreg’ [88] and ‘mgcv’ [38] packages in R. Red lines illustrate predicted relationships from generalized additive models (GAMs) and gray bands denote 95% confidence intervals. Numbers above or below survey years denote sample sizes (i.e., the number of hauls conducted). Effective degrees of freedom (EDF) and individual data points (black ticks along x-axis) are shown for smoothed univariate covariates. (PDF) [file pone.0209402.s002.pdf]

# GOA, 1990 to 2013

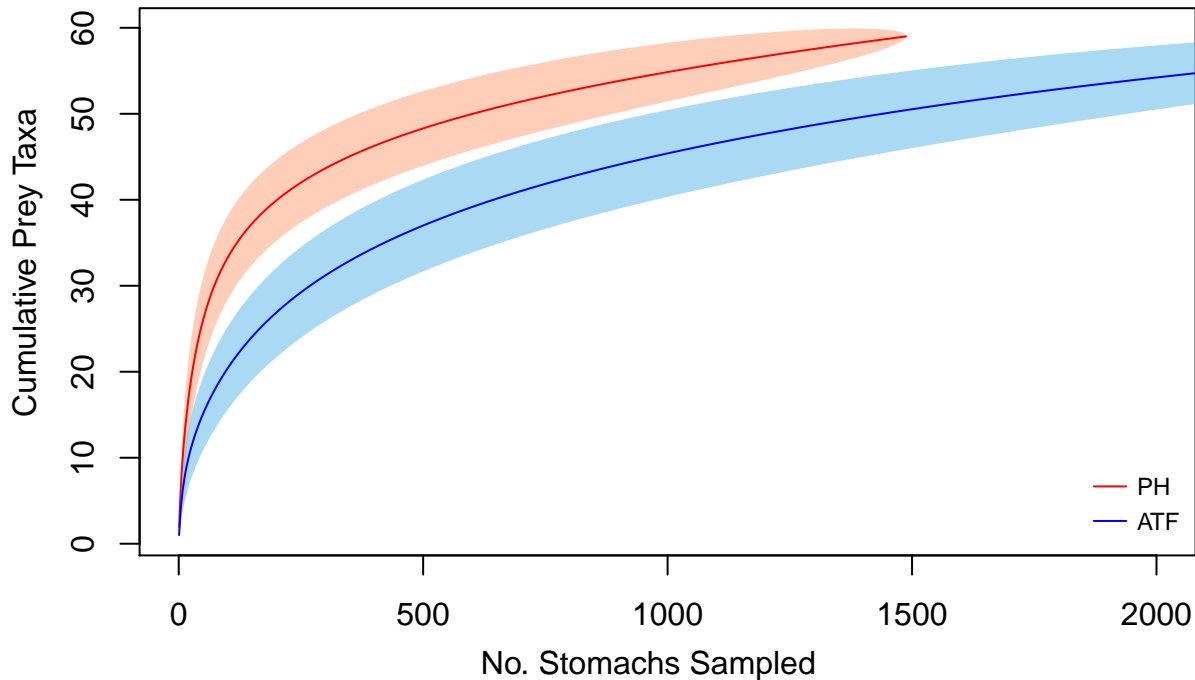

Supplement: S2 Fig — Rarefaction curves illustrating changes in number of cumulative prey taxa encountered with sample size. Pacific Halibut is shown in red and Arrowtooth Flounder is shown in blue. Shaded areas indicate 95% confidence intervals. (PDF) [file pone.0209402.s005.pdf]

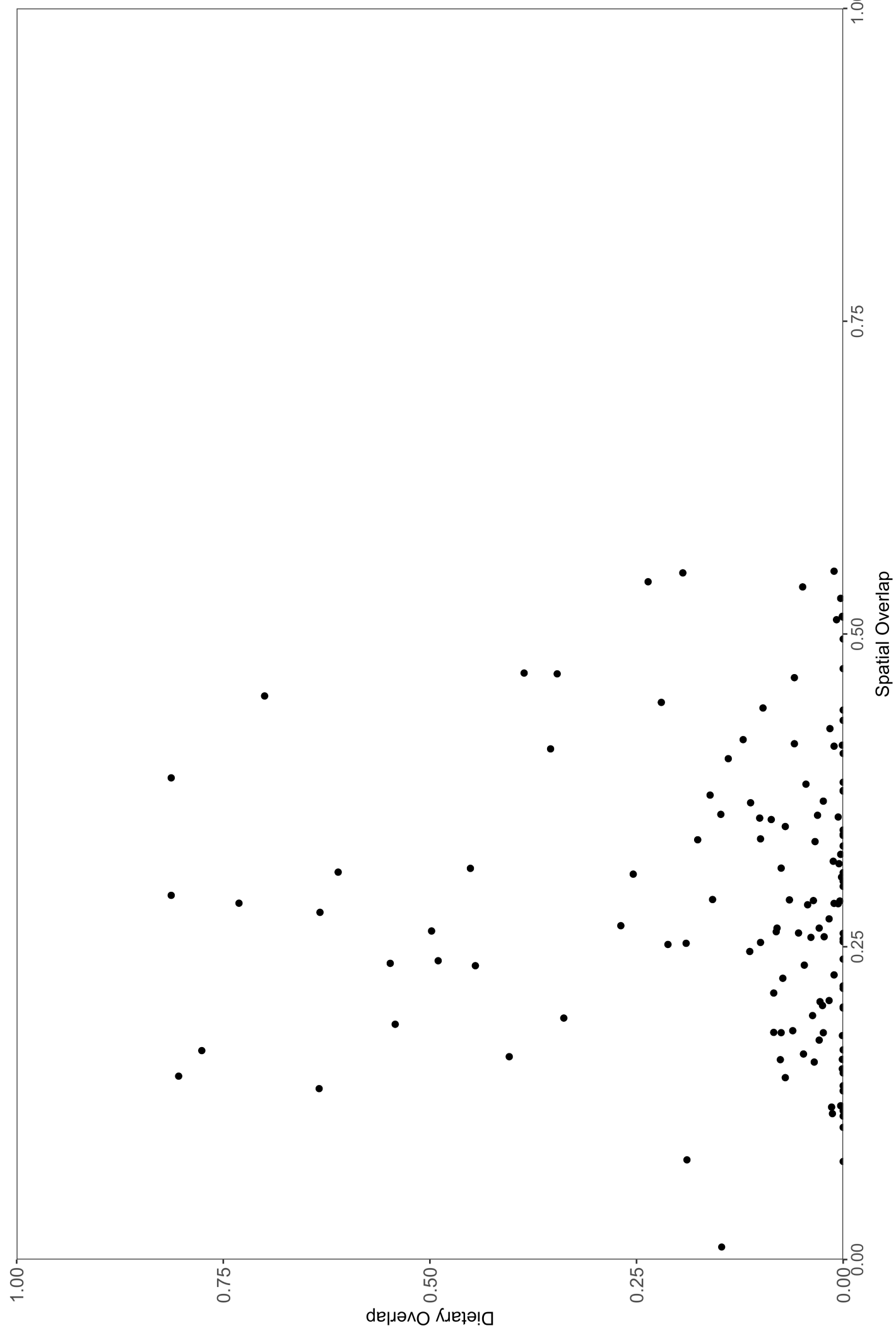

Supplement: S3 Fig — Each data point represents a unique combination of survey year and grid cell. (PDF) [file pone.0209402.s006.pdf]
