# Supplementary material for: Assessing the potential for competition between Pacific Halibut (Hippoglossus stenolepis) and Arrowtooth Flounder (Atheresthes stomias) in the Gulf of Alaska
Source: PLoS One. 2018 Dec 18;13(12):e0209402. doi: 10.1371/journal.pone.0209402 (PMC6298734; doi:10.1371/journal.pone.0209402)
Supplement: S1 Table — Because year was treated as a factor, 1990 is denoted as the model intercept and estimates for subsequent years are shown as differences from 1990. Smoothed variables include location (latitude, longitude), depth, and bottom temperature. Non-significant terms (α = 0.1) are grayed out. (PDF) [file pone.0209402.s003.pdf]

A. Pacific Halibut

Selected GAM	Estimate	Std. Error	z- or t-value	edf	Chi Sq. or F	p-value	Adj. R^2 (scale est.)
**Presence / Absence**							0.514 (1.000)
(intercept)	- 0.158	4.273	- 0.04			0.971	
1993	0.229	0.192	1.19			0.233	
1996	- 0.419	0.193	- 2.17			0.030	
1999	- 0.100	0.196	- 0.51			0.608	
2001	- 0.821	0.215	- 3.81			< 0.001	
2003	- 0.248	0.196	- 1.26			0.206	
2005	- 0.244	0.192	- 1.27			0.205	
2007	0.350	0.199	1.76			0.079	
2009	0.212	0.197	1.08			0.281	
2011	0.545	0.200	2.73			0.006	
2013	0.346	0.206	1.68			0.093	
2015	0.990	0.198	5.00			< 0.001	
2017	0.458	0.205	2.24			0.025	
Lon, Lat				25.8	346.4	< 0.001	
Depth				6.4	1123.3	< 0.001	
Bottom Temp				2.9	15.7	0.0031	
**CPUE** **(no. per ha)**							0.459 (0.963)
(intercept)	4.931	0.106	46.64			< 0.001	
1993	0.401	0.116	3.45			< 0.001	
1996	0.336	0.119	2.83			0.005	
1999	0.317	0.119	2.66			0.008	
2001	0.157	0.123	1.28			0.201	
2003	0.551	0.117	4.73			< 0.001	
2005	0.534	0.116	4.61			< 0.001	
2007	0.558	0.117	4.79			< 0.001	
2009	0.716	0.117	6.14			< 0.001	
2011	0.549	0.116	4.73			< 0.001	
2013	0.385	0.118	3.26			0.001	
2015	0.780	0.115	6.77			< 0.001	
2017	0.459	0.118	3.88			< 0.001	
Lon, Lat				26.0	12.4	< 0.001	
Depth				7.8	210.8	< 0.001	
Bottom Temp				3.0	7.8	< 0.001	

B. Arrowtooth Flounder

Selected GAM	Estimate	Std. Error	z- or t-value	edf	Chi Sq. or F	p-value	Adj. R^2 (scale est.)
**Presence / Absence**							0.383 (1.000)
(intercept)	0.039	0.160	0.24			0.808	
1993	1.811	0.202	8.97			< 0.001	
1996	2.604	0.214	12.15			< 0.001	
1999	3.351	0.231	14.48			< 0.001	
2001	2.431	0.234	10.37			< 0.001	
2003	3.041	0.215	14.14			< 0.001	
2005	3.930	0.231	17.01			< 0.001	
2007	3.065	0.223	13.73			< 0.001	
2009	3.694	0.234	15.77			< 0.001	
2011	3.904	0.244	15.97			< 0.001	
2013	3.231	0.237	13.64			< 0.001	
2015	3.195	0.226	14.12			< 0.001	
2017	3.756	0.257	14.62			< 0.001	
Lon, Lat				28.3	574.3	< 0.001	
Depth				8.0	1113.3	< 0.001	
Bottom Temp				1.0	1.1	0.294	
**CPUE (no. per ha)**							0.399 (1.829)
(intercept)	7.896	0.107	73.75			< 0.001	
1993	- 0.283	0.120	- 2.35			0.019	
1996	- 0.470	0.120	- 3.93			< 0.001	
1999	- 0.388	0.120	- 3.22			0.001	
2001	- 0.452	0.129	- 3.49			< 0.001	
2003	0.003	0.120	0.03			0.978	
2005	- 0.033	0.118	- 0.28			0.782	
2007	- 0.283	0.121	- 2.34			0.019	
2009	- 0.211	0.120	- 1.76			0.079	
2011	- 0.285	0.121	- 2.35			0.019	
2013	- 0.728	0.124	- 5.86			< 0.001	
2015	- 0.349	0.121	- 2.90			0.004	
2017	- 0.622	0.124	- 5.03			< 0.001	
Lon, Lat				28.3	48.7	< 0.001	
Depth				8.1	299.7	< 0.001	
Bottom Temp				2.9	12.2	< 0.001	
