# Supplementary material for: Assessing the potential for competition between Pacific Halibut (Hippoglossus stenolepis) and Arrowtooth Flounder (Atheresthes stomias) in the Gulf of Alaska
Source: PLoS One. 2018 Dec 18;13(12):e0209402. doi: 10.1371/journal.pone.0209402 (PMC6298734; doi:10.1371/journal.pone.0209402)

standardized by predicted max

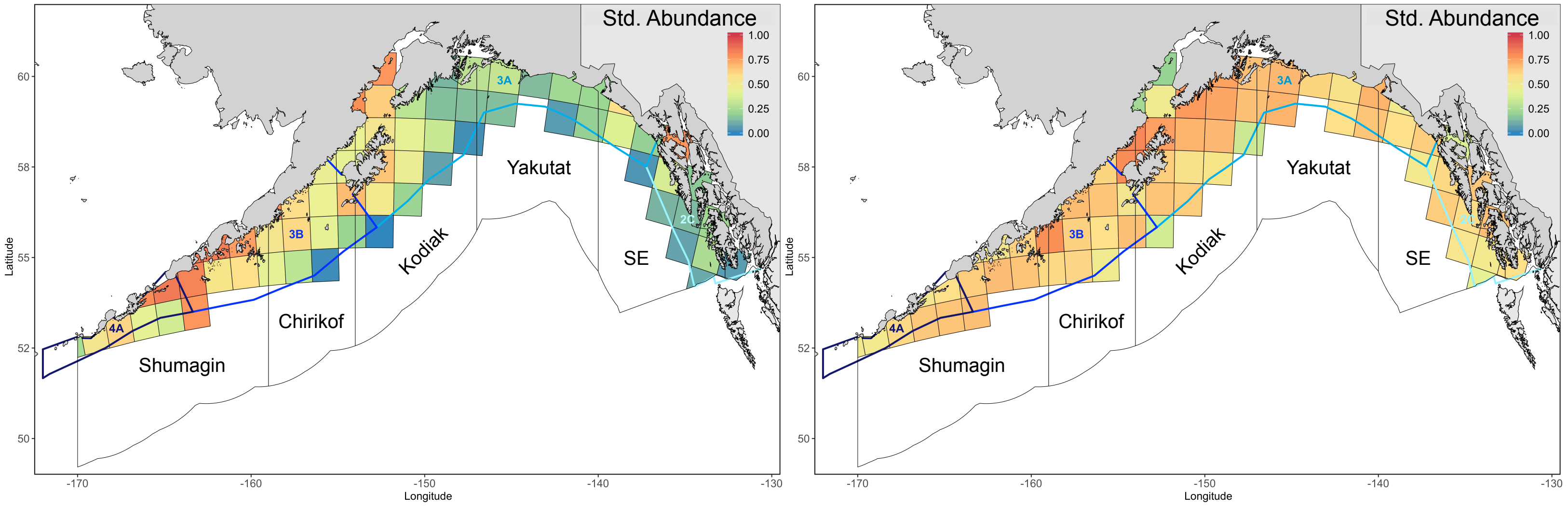

standardized by predicted mean

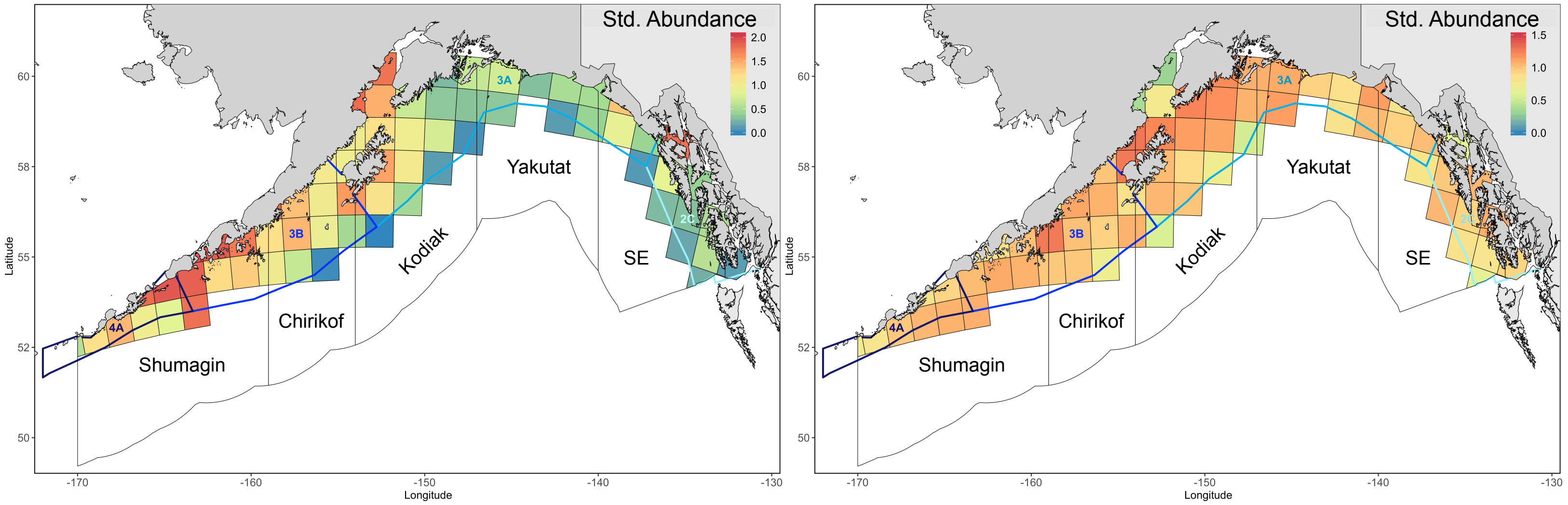

standardized by predicted median

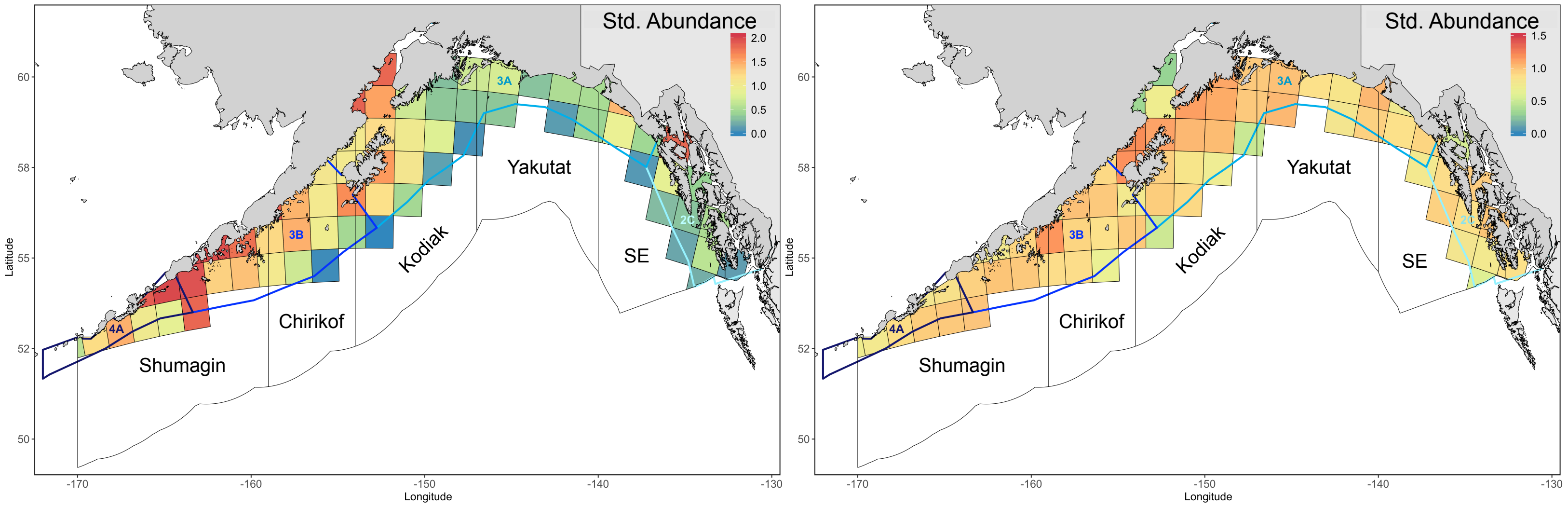

Supplement: S1 Fig — Mean grid cell-specific estimates of abundance (1990 to 2017) for Pacific Halibut (left) and Arrowtooth Flounder (right) using different standardization methods (i.e., dividing individual grid cell abundances by the species-specific maximum, mean, or median predicted abundance). (PDF) [file pone.0209402.s004.pdf]
